# scSNV: accurate dscRNA-seq SNV co-expression analysis using duplicate tag collapsing

**DOI:** 10.1186/s13059-021-02364-5

**Published:** 2021-05-07

**Authors:** Gavin W. Wilson, Mathieu Derouet, Gail E. Darling, Jonathan C. Yeung

**Affiliations:** 1 Latner Thoracic Surgery Research Laboratories, University Health Network, 101 College St., 2-501, Toronto, ON M5G 2C4 Canada; 2 Division of Thoracic Surgery, Department of Surgery, University of Toronto, Toronto, M5G 2C4 Canada; 3 Toronto General Hospital, 200 Elizabeth St, 9N-983, Toronto, ON M5G 2C4 Canada

**Keywords:** Single-cell RNA-seq, Genetic variation, Alignment, Variant calling

## Abstract

Identifying single nucleotide variants has become common practice for droplet-based single-cell RNA-seq experiments; however, presently, a pipeline does not exist to maximize variant calling accuracy. Furthermore, molecular duplicates generated in these experiments have not been utilized to optimally detect variant co-expression. Herein, we introduce scSNV designed from the ground up to “collapse” molecular duplicates and accurately identify variants and their co-expression. We demonstrate that scSNV is fast, with a reduced false-positive variant call rate, and enables the co-detection of genetic variants and A>G RNA edits across twenty-two samples.

**Supplementary Information:**

The online version contains supplementary material available at 10.1186/s13059-021-02364-5.

## Background

Droplet-based single-cell RNA-seq (dscRNA-seq) has become a powerful tool to profile the transcriptome of thousands of single cells in an experiment. Currently, methods to fully exploit the potential of dscRNA-seq data and gain additional biological insights continue to be developed. One such method is the integration of single nucleotide variation (SNV) data to facilitate the study of genetic subpopulations at the transcriptional level [1], the demultiplexing of pooled samples using natural genetic variation between samples [2], and the exploration of expression quantitative trait loci [3]. However, these methods require the accurate and efficient identification of single nucleotide variants (SNVs) from dscRNA-seq data.

**Fig. 1:**
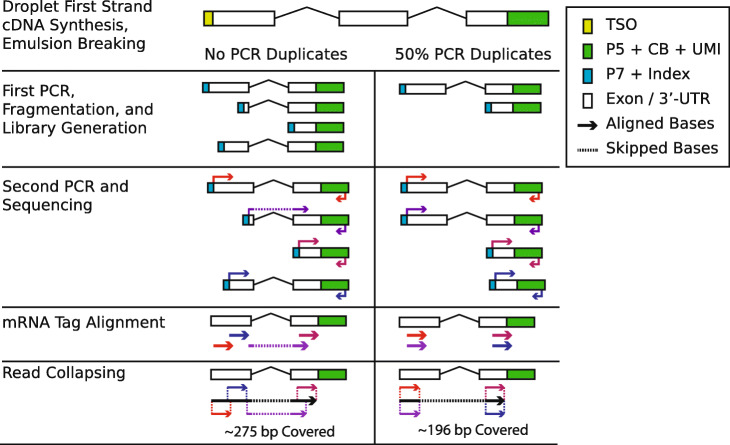
Schematic representing two extreme scenarios for tagged reads generated from the same molecule without any PCR duplicates (left) and with PCR duplicates (right). Row 1: the full cDNA sequence after reverse transcription; row 2: the cDNA molecules after the first round of PCR amplification, fragmentation, and adapter ligation; row 3: the fragmented products after a second round of PCR with arrows indicating the mRNA tags and CB + UMI reads; row 4: the sequencing reads after alignment; and row 5: collapsing of the aligned mRNA tags into long collapsed molecules. Each colored arrow represents the sequencing tag and the cell barcode

A key innovation of dscRNA-seq experiments is the use of three molecular identifiers: (1) the cellular barcode (CB), which is unique to each droplet; (2) the unique molecular identifier (UMI) that is unique to each molecule within a droplet; and (3) the mRNA tag (used interchangeably with read) used to identify the molecules originating transcript [4, 5]. dscRNA-seq library generation consists of four major steps: (1) within droplet reverse transcription and template switching, which integrates the CB and UMI and generates full-length cDNA molecules; (2) full-length cDNA amplification; (3) random fragmentation, size selection, and adapter ligation; and (4) library amplification and sequencing (Fig. 1). The critical observation with the design of these libraries is that random fragmentation occurs after the first round of PCR amplification, which generates sets of cDNA molecules with identical CBs, UMIs, and originating transcripts, but the read sequences can map to multiple unique positions within the transcript (or gene). Subsequently, these fragmented cDNA molecules are amplified a second time leading to a subset of molecules having the same CB, UMI, originating transcript, and mapping position. The resulting fragments are sets of molecular duplicates (identical CB, UMI, originating transcript), but the variability in the reads from the first round of amplification leads to the potential to cover additional bases and SNVs within the originating transcript. The subset of PCR duplicates from the second round of amplification will instead always cover the same bases per read. In practice, the additional bases covered are limited by the size selection step prior to the second PCR round. Nonetheless, this leads to the novel idea to “collapse” molecular duplicates into longer consensus molecules that will provide additional information, such as SNV co-expression.

The molecular duplicate rate is commonly referred to as the sequencing saturation and is calculated by dividing the number of molecular duplicates by the total number of countable reads. Sequencing saturation is an important metric for determining if a dscRNA-seq library has been sequenced to sufficient depth as a high saturation rate implies that additional sequencing will yield fewer new CB, UMI, and read combinations but will instead lead to additional molecular duplicates. Furthermore, it would be beneficial to explore PCR duplicate rate as a metric to assess the quality of a dscRNA-seq library as a high PCR duplicate rate may limit the base coverage of each cell and indicate that the experimental protocol needs optimization, or the sample library complexity was low.

RNA-seq and by extension dscRNA-seq experiments will contain germline SNVs, A>G edits, and technical errors from library generation, sequencing, and alignment [6]. Current dscRNA-seq alignment pipelines, such as Cell Ranger [5], STARsolo (unpublished), Alevin [7], and Kallisto BUStools [8], have focused on gene expression quantification. Kallisto BUStools and Alevin are magnitudes faster than Cell Ranger; however, the aforementioned tools do not produce the full read alignments necessary for SNV calling (Alevin and Kallisto BUStools) or have not been designed to mitigate common sources of false-positive SNVs (Cell Ranger, STARsolo). Unlike low-throughput scRNA-seq methods such as SMART-seq2, which can generate millions of reads per cell, dscRNA-seq data is sparse and has only thousands to tens of thousands of molecules per cell (after duplicate removal) [9]. Due to this issue, SNVs for dscRNA-seq data are typically called on pseudo-bulk samples and quantified in the individual cells [10, 11]. However, this method limits the detection of subclonal SNVs as they will have low minor allele fractions compared to clonal SNVs. Thus, while minimal filtering is necessary when calling SNVs to identify these rare SNVs, it is also likely to increase the false-positive rate. Finally, there is a need for a fast and carefully benchmarked tool to pileup dscRNA-seq data using the cell barcode and duplicate data.

In this work, we present a new dscRNA-seq alignment tool, scSNV, that is designed from the ground up to take advantage of the molecular duplicates produced by dscRNA-seq data by collapsing duplicate reads into longer molecules post-alignment, facilitates the accurate identification of SNVs from dscRNA-seq data, and extracts the co-expression of SNVs present within collapsed molecules. Furthermore, scSNV emits rich metrics for each cellular barcode including the read alignment rates, saturation, PCR duplicate rates, and the other useful metrics to determine the cell barcodes, which are likely to represent cells. For read alignment, we benchmark scSNV against Cell Ranger, the most popular tool used for the analysis of 10X Chromium dscRNA-seq data, and STARsolo, an unpublished alternative to Cell Ranger. For SNV calling, we used a SNV caller built into scSNV, “scSNV Pileup,” and two published SNV callers Strelka2 [12] and samtools/bcftools [13]. We utilized a set of 22 samples (17 public and 5 produced for this study, nine with matched whole genome sequencing and two with matched exome sequencing, see Additional file 1: Table S1 to validate the accuracy of scSNV). Furthermore, we demonstrate that the true-positive rate in single cells is adversely affected by the sparsity of dscRNA-seq experiments using WGS matched scRNA-seq data and simulated data. Overall, we demonstrate that our alignment strategy, duplicate read collapsing, and SNV calling tools reduce the false-positive rate of SNV calling for dscRNA-seq data and that the collapsing strategy permits the identification of multiple edits and/or SNVs in a single molecule.

## Results

### Design of scSNV: a pipeline for molecular duplicate collapsing and SNV calling from dscRNA-seq data

We designed scSNV to facilitate the accurate identification of SNVs from dscRNA-seq data. The current gold standard alignment method for 10X dscRNA-seq data is Cell Ranger [5] using STAR [14] for its underlying alignment, which has a high false-positive mismatch rate [15]. Recently, STAR has added its own unpublished support for dscRNA-seq processing named STARsolo, which is designed to be a faster alternative to Cell Ranger with an almost similar identical output. We chose not to compare our method to pseudoalignment-based tools such as Alevin, as these do not produce full alignments for SNV calling and their quantification estimates have been shown to be identical to Cell Ranger [7].

We pre-filtered each alignment read to remove those with low sequence complexity and used BWA-mem [16] to map each read to the genome to identify intergenic and unspliced reads and to the transcriptome to identify spliced reads (see “Methods” section). The transcript alignments are projected to genomic coordinates, and the alignments from both are trimmed to eliminate alignment artifacts from incorrectly mapped reads containing exon-exon junctions, which have previously been shown to generate false-positive SNVs [15, 17]. We were stringent with the identification of multi-mapped reads by including both intergenic and genic alignments, which differs from Cell Ranger, which ignores intergenic alignments if one of the alternative alignments map to a genic region. The transcript and genomic alignments are merged to identify the highest scoring alignments, which are retained for further analyses. These alignments are deduplicated using the directional UMI algorithm, where spliced and unspliced molecular counts are calculated.

### Benchmarking the run time of scSNV and quantification accuracy versus Cell Ranger and STARsolo

We used twenty-two publicly available datasets including 9 normal tissue samples [18], 3 blood-derived samples (10X Genomics), 5 pancreatic ductal adenocarcinoma (PDAC) samples [19] for benchmarking, and an additional 5 esophageal adenocarcinoma organoid (EAC) samples we generated for this study (Additional file 1: Table S1). The normal tissues have matched whole genomes and two of the EAC samples have matched exomes. For each of the twenty-two samples included in this study, we processed the raw sequencing data with scSNV, Cell Ranger, and STARsolo. First, we explored the run time of scSNV (including read collapsing) to Cell Ranger and found that scSNV was 2.68±0.89 times faster than Cell Ranger, STARsolo was 3.98 ±1.53 times faster than scSNV, and STARsolo was 10.25±4.46 times faster than Cell Ranger (Additional file 2: Figure S1). The low performance of Cell Ranger is likely due to splitting reads by barcode, multiple file passes, and parts of its code being implemented in Python. Both scSNV and STARsolo are implemented in C++ and minimize the amount of data written to disk and the number of times files are processed. The reason STARsolo was faster than scSNV was likely due to STAR being faster than BWA-mem and the additional read collapsing step (8.07 ± 2.56% of runtime) implemented as part of scSNV requiring a second pass of the bam file and writing an additional bam file to disk.

To validate that our alignment scheme produces similar spliced and unspliced molecular counts as Cell Ranger with velocyto, we used eleven of our samples and found similar total spliced molecules and a high Spearman correlation for the spliced and unspliced counts for each barcode (average median for spliced = 0.856 ± 0.012 and unspliced = 0.875 ± 0.022) (Additional file 2: Figure S2). However, for unspliced molecular counts, we observed a drop in the Spearman correlation for cells with low intronic molecular counts. This may be due to our high stringency for the detection of multi-mapped reads and our inclusion of additional gene biotypes that are not included in Cell Ranger’s reference. We did not compare our results to STARsolo as its spliced and unspliced read quantification is still in development but it is designed to emit similar counts as Cell Ranger and velocyto. We have demonstrated that despite our stringent alignment steps, which involved the removal of common sources of false-positive SNVs, we emit similar quantification values that can be used as an alternative to Cell Ranger and STARsolo.

### Collapsing molecular and PCR duplicates yields longer molecules

**Fig. 2:**
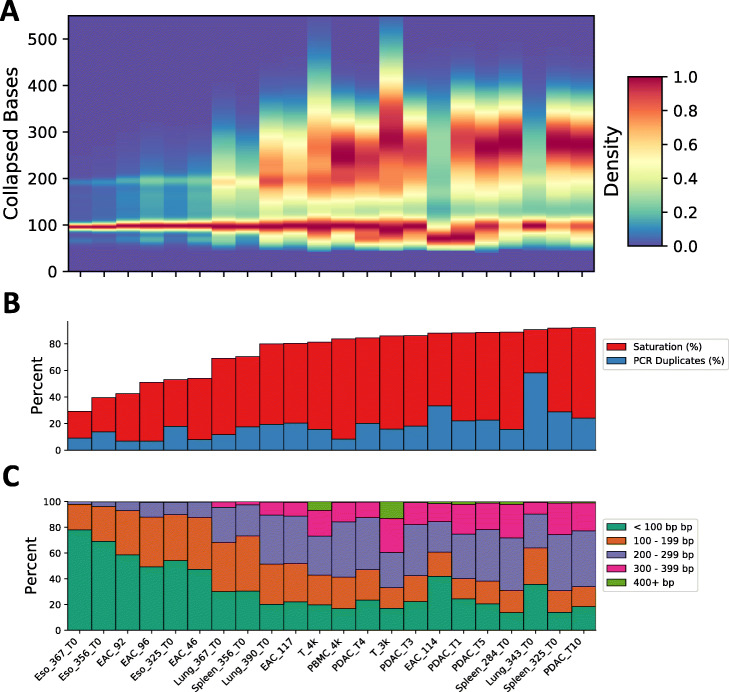
The effect of sequencing saturation and the PCR duplicate rate on mRNA tag collapsing. **a** Collapsed molecule lengths from each sample ordered by their sequencing saturation (lowest to highest). For each sample, the collapsed read lengths were binned by their length and the resultant distributions were each down-sampled to 20,000 observations, smoothed using Gaussian kernel density estimation, and then scaled so the minimum and maximum value ranged from 0 to 1. The density values are indicated by the color bar. **b** The sequencing saturation (red) and PCR duplicate rates (blue) for each sample. **c** The percent of molecules in each of the collapsed base ranges as indicated by the legend

In the majority of 10X dscRNA-seq experiments, the read/mRNA tag is sequenced to 98 bp; however, the existence of molecular duplicates in each dataset permits the “collapsing” of these reads into consensus sequences which produces longer collapsed molecules (Fig. 2a). To do this, we developed a method that will merge the alignments into longer consensus sequences from countable reads, i.e., those confidently mapped to spliced or unspliced regions. These collapsed molecules are emitted as standard BAM files that can be used for downstream analyses. Next, we sought to explore the number of mapped bases in each collapsed molecule in relation to the samples’ overall sequencing saturation and PCR duplicate rate and to the number of underlying duplicate reads used to generate the collapsed molecule. Due to the size selection step after the full-length cDNA is fragmented, we expected that the collapsed molecules would be limited to sizes in the range of 300–600 bp (118 to 418 bp of cDNA). We observed an expected peak at 98 bp that represents collapsed molecules consisting of a single unique read (average 32.91 ± 18.25% of molecules) and that the number of mapped bases in each collapsed molecule was generally limited to <400 bp (average 98.51 ± 2.90% of collapsed molecules) (Fig. 2b, S3). In general, the maximum number of collapsed bases in a molecule was limited by the number of duplicate reads in the molecule; for example, with three reads, the maximum size was 296 bp (Additional file 2: Figure S3). We also observed that the number of collapsed bases in a molecule was dependent on the sample’s sequencing saturation, where a higher sequencing saturation led to more collapsed bases (Fig. 2c). The effect of increased saturation was limited by the PCR duplication rate, where a high PCR duplicate rate negated the effect for increased saturation by decreasing the collapsed molecule sizes compared to samples with similar saturations. Thus, we have demonstrated that the information gained from collapsing duplicate reads is useful to glean more information from a dscRNA-seq experiment and dependent on both sequencing saturation and PCR duplicate rate.

### scSNV has fewer false-positive SNV calls than Cell Ranger and STARsolo when using pseudo-bulk samples

SNVs identified from dscRNA-seq data have been used to identify genetic [1] or to demultiplex pooled samples by the natural genetic variation between samples [2]. Previous evaluation of SNV calling has focused on low-throughput (plate based) single-cell RNA-seq methods such as SMART-seq2 [9, 20] or the Fluidigm C1 instrument [21]. The aforementioned methods sequence the whole transcriptome from hundreds of cells with millions of paired-end reads per cell, which permits SNV calling for each cell independently. The aforementioned studies have not extensively profiled droplet-based scRNA-seq datasets, performed a detailed comparison between the effects of dscRNA-seq alignment algorithms on SNV calling accuracy, examined RNA editing events, nor assessed the effect of dscRNA-seq sparsity on the number of SNVs detected at the single-cell level.

For dscRNA-seq libraries, 3′-tagged (or 5′-tagged) molecules are sequenced from thousands to tens of thousands of cells with tens of thousands of single-end reads per cell (hundreds to thousands of molecules per cell after deduplication). SNVs are then called across the pooled cells by treating their alignments as a “pseudo-bulk” sample since the read coverage in an individual cell is too sparse for accurate SNV calling. Therefore, methods to call rare SNVs (low alternative allele counts in a pseudo-bulk sample) are essential to identify SNVs unique to a given cell type or population, for example, tumor cells in a mixed tumor/normal sample or to deconvolute pooled experiments consisting of multiple unique individuals.

Current standard SNV calling methods are not designed to utilize the strand-specificity and UMI duplicates in dscRNA-seq data and do not extract per-cell barcode allele counts. To overcome these issues, we developed a pileup method available with the scSNV package that filters based on the number of barcodes with coverage, the number of barcodes with an alternative allele at a given base, a minor allele fraction cutoff (to eliminate very rare SNVs), and basic filtering to remove common sources of false positives such as mis-spliced reads (see the “Methods” section). Our pileup method emits sparse SNV count matrices to minimize disk and memory usage and can be used with a pre-existing set of known SNVs to quantify strand-specific and per-barcode SNV counts (per cell).

Our first aim was to determine if scSNV alignment with read collapsing has an effect on SNV calling accuracy compared to Cell Ranger and STARsolo. Currently, a protocol does not exist to simulate dscRNA-seq data with sequencing errors, UMI and PCR duplicates, and alignment artifacts. Therefore, as an alternative, we used nine normal samples (3 lung, 3 spleen, and 3 esophagus) with matched whole genome data and two esophageal cancer organoids with matched exome data. We constrained our analyses to regions covered in the genome sequencing data, and for the two EAC organoids with matched exome data, we further limited our analyses to regions captured by the exome array plus 100 bp of flanking sequences. By limiting our analysis to well-covered regions in the matched DNA sequencing data, we can define true positives (identified in the dscRNA-seq sample and genome data), false positives (called in a dscRNA-seq sample and covered in the genome data with less than 2 alternative base counts), and false negatives (called in the genome and at least one dscRNA-seq samples but missed in another) (see the “Methods” section).

For each dscRNA-seq sample, we used our pileup on the pooled alignments produced by scSNV with collapsing (“scSNV Collapsed”) and without (“scSNV Tags”), Cell Ranger, and STARsolo to identify SNVs. As an additional comparison, we called SNVs for each of the four alignment methods using the two SNV callers recommended by Liu et al. [20]: samtools/bcftools [13] and Strelka2 [12]. When calling scSNV from Cell Ranger and STARsolo, we used all reads marked as duplicates as these include both UMI and PCR duplicates and discarding these negatively affected SNV calling performance (data not shown). When calling SNVs on alignments from scSNV without read collapsing, we properly mark and discard PCR duplicates while retaining UMI duplicates. It should be noted that Strelka2 does not provide an option to include reads marked as PCR duplicates. Cell- and strand-specific data was extracted using our pileup method with filtering disabled to extract strand- and barcode-specific allele counts for barcodes that were marked as cells by scSNV and to select for bi-allelic and strand-specific SNVs (see the “Methods” section). Finally, we applied minimal filtering to all of the SNV calls by requiring coverage by 5 or more barcodes and 2 barcodes with the alternative allele to remove SNVs called from reads that were not marked as cells.

To begin, we examined the true-positive rate (TPR), false discovery rate (FDR), and F1 score. For these analyses, we identified potential A>G edits as strand-specific A>G SNVs found in the REDIportal database or within Alu [22]; we then separated these sites from the other SNVs and examined them separately. First, we observed that the TPR was highest using scSNV pileup with all four alignment methods with a TPR ranging from 0.918 to 0.954 (Table 1, Additional file 1: Table S2), followed by samtools/bcftools with a range of 0.778–0.870, and Strelka2 with a range of 0.021 to 0.143. We further observed that Strelka2 had a low FDR among all of the samples (<0.05) and F1 score (<0.205).

**Table 1:** Accuracy metric summaries for alignment and SNV calling algorithms

SNV caller	Metric	scSNV Collapsed	scSNV Tags	Cell Ranger	STARsolo
**scSNV Pileup**	TPR	0.954 ± 0.005	0.953 ± 0.005	0.94 ± 0.011	0.955 ± 0.005
**scSNV Pileup + AF cutoff**	TPR	0.934 ± 0.009	0.918 ± 0.016	0.899 ± 0.012	0.912 ± 0.013
**samtools/bcftools**	TPR	0.819 ± 0.031	0.819 ± 0.03	0.81 ± 0.023	0.821 ± 0.028
**Strelka2**	TPR	0.037 ± 0.014	0.115 ± 0.025	0.11 ± 0.019	0.110 ± 0.02
**scSNV Pileup**	FDR	0.430 ± 0.052	0.547 ± 0.041	0.734 ± 0.055	0.729 ± 0.057
**scSNV Pileup + AF cutoff**	FDR	0.108 ± 0.029	0.109 ± 0.031	0.331 ± 0.038	0.322 ± 0.044
**samtools/bcftools**	FDR	0.049 ± 0.015	0.048 ± 0.014	0.178 ± 0.035	0.152 ± 0.027
**Strelka2**	FDR	0.039 ± 0.019	0.032 ± 0.015	0.052 ± 0.02	0.053 ± 0.018
**scSNV Pileup**	F1	0.713 ± 0.04	0.613 ± 0.038	0.412 ± 0.067	0.419 ± 0.07
**scSNV Pileup + AF cutoff**	F1	0.912 ± 0.017	0.904 ± 0.021	0.767 ± 0.028	0.777 ± 0.03
**samtools/bcftools**	F1	0.880 ± 0.021	0.880 ± 0.020	0.816 ± 0.025	0.834 ± 0.021
**Strelka2**	F1	0.071 ± 0.026	0.204 ± 0.041	0.197 ± 0.031	0.196 ± 0.032

As demonstrated above, our Strelka2 results using pseudo-bulk samples were inconsistent with the findings of Liu et al. [20] where they observed reasonable accuracy for Strelka2 compared to samtools/bcftools when calling simulated homozygous SNVs on individual cells from a single 10X dscRNA-seq experiment. Moreover, we examined the SNV calls that failed Strelka2’s default filtering parameters and observed that 3.66 ± 1.28% of the filtered calls were true positives from our whole genome sequencing data and 91.2% of those were filtered due to a low GQX score. This GQX score is derived from a random-forest classifier trained on features extracted from SNVs called on full-length paired-end RNA-seq data; however, in contrast, dscRNA-seq data is 3′-end biased, strand-specific, and single-end [12]. Therefore, Strelka2 is not ideal for SNV calling on pseudo-bulk dscRNA-seq samples.

For the remainder of this section, we focus on the TPR and FDR for SNVs called with scSNV pileup and samtools/bcftools. We observed that SNVs called with scSNV pileup had a higher FDR than SNVs called using samtools/bcftools; however, for each sample alignment, Cell Ranger had the highest FDR, followed by STAR, scSNV Tags, and scSNV Collapsed (Table 1). Finally, we found that for scSNV Collapsed and scSNV Tags, Cell Ranger and STARsolo identified similar numbers of A>G edits with scSNV Pileup (>80% of the union of all A>G edits detected in a given sample by each of the alignment and SNV calling methods) and identified many fewer edits using samtools/bcftools (<25% of detected edits). From this, we can conclude that alignments with scSNV with or without collapsing elicits a reduction in the FDR using both SNV calling methods and that there appears to be a trade-off for the TPR and FDR and for A>G edit identification when calling SNVs using samtools/bcftools.

### The majority of false positives detected by scSNV have low minor allele fractions

**Fig. 3:**
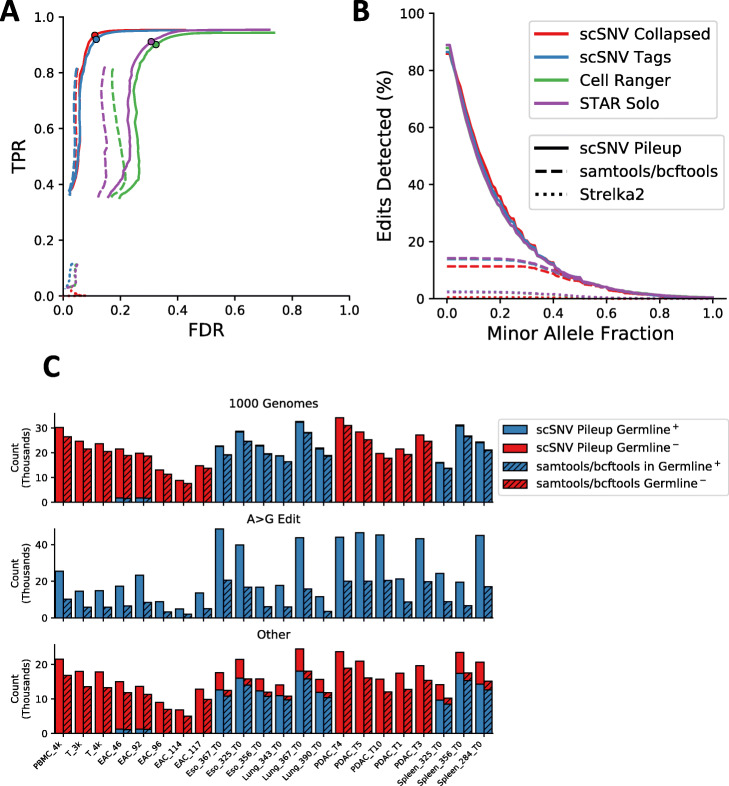
Comparison of SNV calling performance using four alignment methods and three SNV calling methods. **a** The median TPR and FDR rates stratified by increasing minor allele fraction cutoffs estimated using matched DNA sequencing. The color indicates the alignment method and line style indicates the SNV calling method as per the legend. The dots on the lines using scSNV Pileup indicate the position of our recommended minor allele fraction of 0.25. **b** The number of A>G edits detected with increasing minor allele fraction cutoffs using the same methods as **a**. **c** The number of SNV calls (in thousands) for SNVs overlapping 1000 Genomes common SNVs (first row), predicted to be A>G edits (second row), or SNVs without an overlapping annotation (third row) for SNVs identified using scSNV Collapse with scSNV Pileup (solid) or samtools/bcftools (dashed) across twenty-two samples. For samples with matched DNA sequencing data, the number of SNVs found within the germline calls for each method is indicated in blue, while those not found in the germline calls are indicated in red

Since we observed that the FDR is high for SNVs called using scSNV Pileup versus samtools/bcftools, we sought to explore if additional filtering could reduce the FDR as we only applied minimal filtering when calling SNVs. Therefore, we investigated the effect of overall minor allele fraction cutoffs (total alternative bases/total bases summed from all of the cells) using each of the alignment tools. We observed that the FDR dropped substantially, the F1 score peaked, and the TPR had a small reduction for all three alignment methods when a minor allele fraction cutoff of >0.25 was used (Additional file 2: Figure S4). This is in contrast to samtools/bcftools where the FDR did not decrease with increased allele fraction cutoff. When the TPR is compared to the FDR, we observe an “elbow” where the FDR is substantially decreased for each alignment tool, while the TPR remains nearly the same (Fig. 3a, Additional file 2: Figure S5). For A>G edits, this was not the case and the number of edits was substantially reduced with increased allele fraction cutoffs; therefore, for analyses pertaining A>G edits, we do not recommend any additional SNV filtering (Fig. 3b, Additional file 2: Figure S6). For each sample, we identified the “elbow” of the TPR vs FDR plots: 0.269 ± 0.037 for scSNV Collapsed, 0.243 ± 0.025 for scSNV Tags, 0.267 ± 0.176 for Cell Ranger, and 0.274 ± 0.024 for STARsolo (Additional file 2: Figure S5). Therefore, we applied an allele fraction cutoff of 0.25 to the SNVs called using scSNV Pileup and found this substantially reduced FDR with a minor decrease in TPR for each alignment method, scSNV Collapsed (−0.033±0.013 TPR and −0.334 ± 0.56 FDR), scSNV Tags (−0.036 ± 0.015 TPR and −0.441 ± 0.036 FDR), Cell Ranger (−0.053 ± 0.016 TPR and −0.419 ± 0.036 FDR), and STARsolo (−0.057 ± 0.013 TPR and −0.427 ± 0.037 FDR) (Table 1, Additional file 1: Table S2). We also observed that the TPR and FDR scores were similar for samples constrained to the whole genome sequencing data and the two samples constrained to regions captured by the exome data (Additional file 2: Figure S5).

Finally, with our newly adopted minor allele fraction cutoff, we sought to explore the effect of relaxing our genomic coverage constraint and using all twenty-two of our samples. For these, we only used scSNV collapsed alignments as we have previously demonstrated a reduction in the number of false-positive SNV calls. Since we cannot identify germline SNVs, we instead used common SNVs identified from 1000 Genomes [23]. Again, we separated SNVs into three categories, found in 1000 Genomes, A>G edits, and other changes not found in the two previous categories. For comparison, we included the proportion of SNVs identified in the matched exome or whole genome sequencing for the eleven samples with matched data. First, we observed that the majority of SNVs called in matched genome sequencing was found in 1000 Genomes (98.86 ± 0.30 scSNV Pileup and 99.17 ± 0.40 samtools/bcftools). Consistent with our previous observations, we found significantly more SNVs using scSNV Pileup compared to samtools/bcftools in 1000 Genomes, average log_2_ fold change 0.186 ± 0.040, A>G edits, average log_2_ fold change 2.818 ± 0.381, and other SNVs, average log_2_ fold change 0.4 ± 0.053 (*P* < 0.001 for all comparisons using a one-sided Wilcoxon signed-rank test) (Fig. 3c). Interestingly, a large proportion of the other SNVs were found in the matched whole genome sequencing data: 73.72 ± 3.30% with scSNV Pileup and 87.13 ± 2.28% with samtools/bcftools, which suggests that constraining our analyses to SNVs found in 1000 Genomes alone would cause a large reduction in the number of detected germline SNVs. Overall, these results are consistent with our previous accuracy analyses using regions constrained to the whole genome.

In conclusion, we observed that collapsing reads led to a small increase in TPR and decrease in FDR after filtering using scSNV Pileup but had little benefit when using samtools/bcftools. We also observed that the trade-off of using samtools/bcftools compared to scSNV was a reduction in TPR and FDR when Pileup leading to slightly higher F1 scores for alignments with scSNV and slightly lower F1 scores for alignments with STARsolo and Cell Ranger. However, when using samtools/bcftools, the majority of A>G edits are lost. STARsolo also had slightly better performance than Cell Ranger which may partially be due to STARsolo’s inclusion of more genome annotations by default than Cell Ranger, which discards genes of various biotypes. We also observed that scSNV performed better than Cell Ranger and STARsolo due to a substantial reduction in the FDR while maintaining similar TPR’s and numbers of A>G edit calls.

### Profiling the SNV calling accuracy in single cells

Due to the sparsity of dscRNA-seq data, calling SNVs in dscRNA-seq pseudo-bulk samples is currently the only practical way to call SNVs from dscRNA-seq samples. We used scSNV Pileup to quantify strand- and single-cell-specific SNV expression with filtering (see the “Methods” section) for calling with scSNV Pileup and without filtering for callers designed for bulk sample(s), i.e., samtools/bcftools. We define “cell/SNV” as a cell and SNV loci combination for a given cell and SNV loci. For the next three sections, we briefly describe our methodology to profile SNV calling accuracy in single cells. For the analyses, we used the nine samples with matched WGS data and excluded the two samples with matched exome data as they are limited to a constrained region of the genome, which limits the number of true-positive SNVs (Fig. 3c). We limited our benchmarking to alignments with scSNV with collapsing and STARsolo as they produced similar pseudo-bulk SNV calling rates as scSNV without collapsing and Cell Ranger, respectively (Fig. 3a). Furthermore, we only used scSNV Pileup and samtools/bcftools for SNV calling as Strelka2 performed poorly in the pseudo-bulk analyses. For each cell from each of the nine samples, we calculated per-cell accuracy metrics including TPR, FDR, and the number of A>G edits using the SNV calls from their respective pseudo-bulk samples. We aggregated the results from all nine samples yielding 36,370 total cells.

### Profiling the TPR in single cells using matched WGS and simulated dscRNA-seq data

**Fig. 4:**
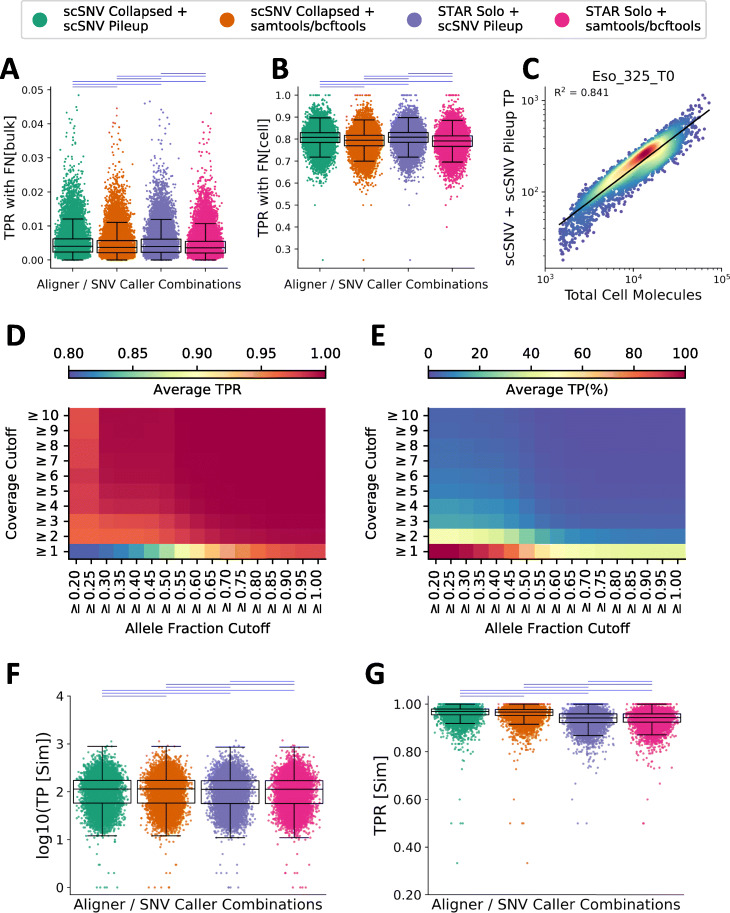
Assessing true-positive detection for single cells using the nine normal tissue samples (*N* = 36,581 cells) with matched WGS data. **a**, **b** The per-cell TPR rate using FN[bulk] (**a**) and FN[cell] (**b**) (see the “Results” section for description). Each aligner/SNV caller is indicated as per the legend and the horizontal lines above the plot indicate the results of a two-sided Wilcoxon signed-rank test (*P* < 0.001 blue otherwise red). **c** Representative comparison of the number of true-positive SNVs versus the number of spliced + unspliced molecules in the cells from Eso_325_T0, the *R*^2^ value and line of best fit as determined by ordinary least squares regression are indicated on the plot (*P*<0.001). **d**, **e** Heatmaps of the average TPR (**d**) and percent of the true positives detected in each cell (**e**) across the cells from all nine samples after applying increasing coverage cutoffs (*y*-axis) and average minor allele fraction cutoffs across the cells from each sample (*x*-axis) for alignments with scSNV and SNV calls using scSNV Pileup (for other tool combinations, see Additional file 2: Figure S7). **f**, **g** The log10 number of true-positive SNVs (**f**) and the TPR (**g**) for the aggregated data from all three simulation experiments, where 25,000 homozygous SNVs were simulated; significant comparisons are indicated as in **a**. The boxplots for **a**, **b**, **f**, and **g** indicate the 25th, 50th, and 75th percentiles and the whiskers are 1.5 times the interquartile range

When profiling the TPR in single cells, two different false-negative (FN) values were calculated for each cell: (1) FN[bulk] a true-positive SNV detected in the pseudo-bulk but with only reference counts or no coverage in a given cell and (2) FN[cell] a true-positive cell/SNV loci with only reference counts (at least 1X coverage). The former is used to demonstrate that dscRNA-seq is sparse and that a given cell will only express a fraction of the SNVs detected in the pseudo-bulk sample, while the second is a subset of the former and the biologically relevant false-negative count that represents SNV loci that are expressed without any detectable alternative allele counts in a given cell. A TP was defined as a cell/SNV with at least one reads/molecule supporting the alternative allele in a given cell. As expected, the median per-cell TPR calculated using FN[bulk] was very low (~0.04 all four tool combinations) compared to the median TPR with FN[cell] (scSNV alignments, 0.808 for scSNV Pileup and 0.794 with samtools/bcftools, and STARsolo alignments, 0.808 for scSNV Pileup and 0.792 samtools/bcftools) and reinforces the sparsity issue of dscRNA-seq data (Fig. 4a, b). The median TPR for each combination was very similar between alignment tools and was slightly higher using scSNV Pileup. Nonetheless, each comparison between the tool combinations was significantly different (*P* < 0.01 two-sided Wilcoxon signed-rank test) despite a high Pearson *R*^2^ value between each combination (>0.92 *P* < 0.01). Finally, we sought to explore the relationship between the number of true-positive (TP) SNVs and the total molecules assigned to each cell and found a high *R*^2^ correlation (0.85 ± 0.05) across all nine samples using the TP determined by scSNV + scSNV Pileup and the total spliced + unspliced molecules by scSNV (ordinary least squares regression) (Fig. 4c for an example sample).

This demonstrates that germline (TP) SNVs can indeed be profiled at the single-cell level in dscRNA-seq samples; however, a given cell only expresses a very low fraction of the total SNVs detected in a pseudo-bulk sample due to the sparsity of dscRNA-seq data.

Despite the reasonable TPR when using false negative (FN)[cell], the results were still lower than the SNV calling results suggesting that a large number of SNVs are missed at the single-cell level. We reasoned that this lower TPR rate is due to a combination of the low per-cell coverage of dscRNA-seq data and the presence of heterozygous SNVs in the genome. For example, a heterozygous SNV with a coverage of one in a given cell could support the alternative allele (TP) or the reference allele (FN). Therefore, we profiled the average TPR from all of the cells using multiple combinations of minor allele fraction (average minor allele fraction across all of the cells from a given sample) and coverage cutoffs. We observed that the average TPR approaches 1.0 as the allele fraction approaches 1.0 (homozygous SNVs) or the coverage approaches 10 (Fig. 4d, e, Fig S7). Moreover, we observed that the coverage cutoff was shifted to higher numbers for alignments with STARsolo. We suspected that this was due to the inclusion of molecular and PCR duplicates for STARsolo SNV calling. Indeed, when we compared the log2 fold change in the coverage of each cell/SNV combination versus the saturation for SNVs with at least 5X coverage (Additional file 2: Figure S8), we observed that the average log2 fold change increased with saturation (0.55 ± 0.42 for Eso_325_T0, saturation 29.2%) and (1.97 ± 0.78 for Spleen_325_T0, saturation 91.9%) and for each sample the difference in coverage was significant (*P* < 0.001 two-sided Wilcoxon signed-rank test). In conclusion, this data suggests that the reduction in TPR at the single-cell level is due to heterozygous SNVs and low coverage.

We have now demonstrated that the TPR is reduced due to the sparsity of dscRNA-seq data and the presence of heterozygous SNVs. Since we cannot distinguish whether a TP cell/SNV is missed due to an alignment or SNV calling issue or due to the sparsity of dscRNA-seq data, a simulation is necessary to further validate each of the benchmarked methods’ accuracy. As a method to simulate dscRNA-seq reads with UMIs, 3′-end bias, etc. does not currently exist, we adopted a modified reference genome-based simulation method for three of the nine WGS matched dscRNA-seq samples similar to the method used to profile the SMART-seq2 data [20]. For each of the three samples, we randomly generated 25,000 base substitutions in the reference genome sequence at positions with at least 5X coverage from scSNV or STARsolo in the unmodified genome (see the “Methods” section). Each of these simulated SNVs will be homozygous, and their detection will no longer be dependent on their minor allele fraction or coverage. In other words, if there is coverage at a given cell/SNV, it should be detected unless there are alignment errors, or the site is missed by the SNV caller. When calculating FN’s, we used both the coverage from the modified genome and the unmodified genome to capture cases where coverage at a particular cell/SNV was lost due to alignment errors from the simulation. For example, if the simulated SNV is the end of a read, it may be partially mapped by the aligner in the modified genome, but completely mapped across the SNV loci in the unmodified genome.

We detected 94.2–96.8% of the 25,000 simulated SNVs with scSNV alignments and 91.0–96.6% of the SNVs with STARsolo alignments for the pseudo-bulks of all three samples. As expected, we observed that each cell only expressed a fraction of the simulated SNVs (median of 113–115 SNV’s per cell for each tool combination) (Fig. 4f). However, the median TPR was very high (scSNV alignments with 0.969 scSNV Pileup, samtools/bcftools 0.966; STARsolo alignments with scSNV Pileup 0.942 and samtools/bcftools 0.943) (Fig. 4g). Despite the very similar results, each of the comparisons was significantly different (*P* < 0.01 two-sided Wilcoxon signed-rank test). The TPR was slightly higher for SNVs called with scSNV for alignment and very similar between SNV calling methods. This simulation study validates that all four of the tool combinations have sensitive detection of homozygous SNVs within single-cells when called from a pseudo-bulk analyses, i.e., if a cell expresses a homozygous SNV, they are likely to be detected by all of the benchmarked methods.

Collectively, we have demonstrated that each aligner/SNV caller combination performed well for the detection of true-positive (germline or simulated) SNVs from pseudo-bulk samples; however, the sparsity (low coverage) of dscRNA-seq data limits the detection of non-homozygous SNVs such as heterozygous SNVs in single cells. Finally, scSNV alignments have a slightly higher true-positive SNV detection rate compared to STARsolo and SNV calling between scSNV Pileup and samtools/bcftools is very similar.

### Alignments with scSNV have a reduced FDR compared to STARsolo in single cells

**Fig. 5:**
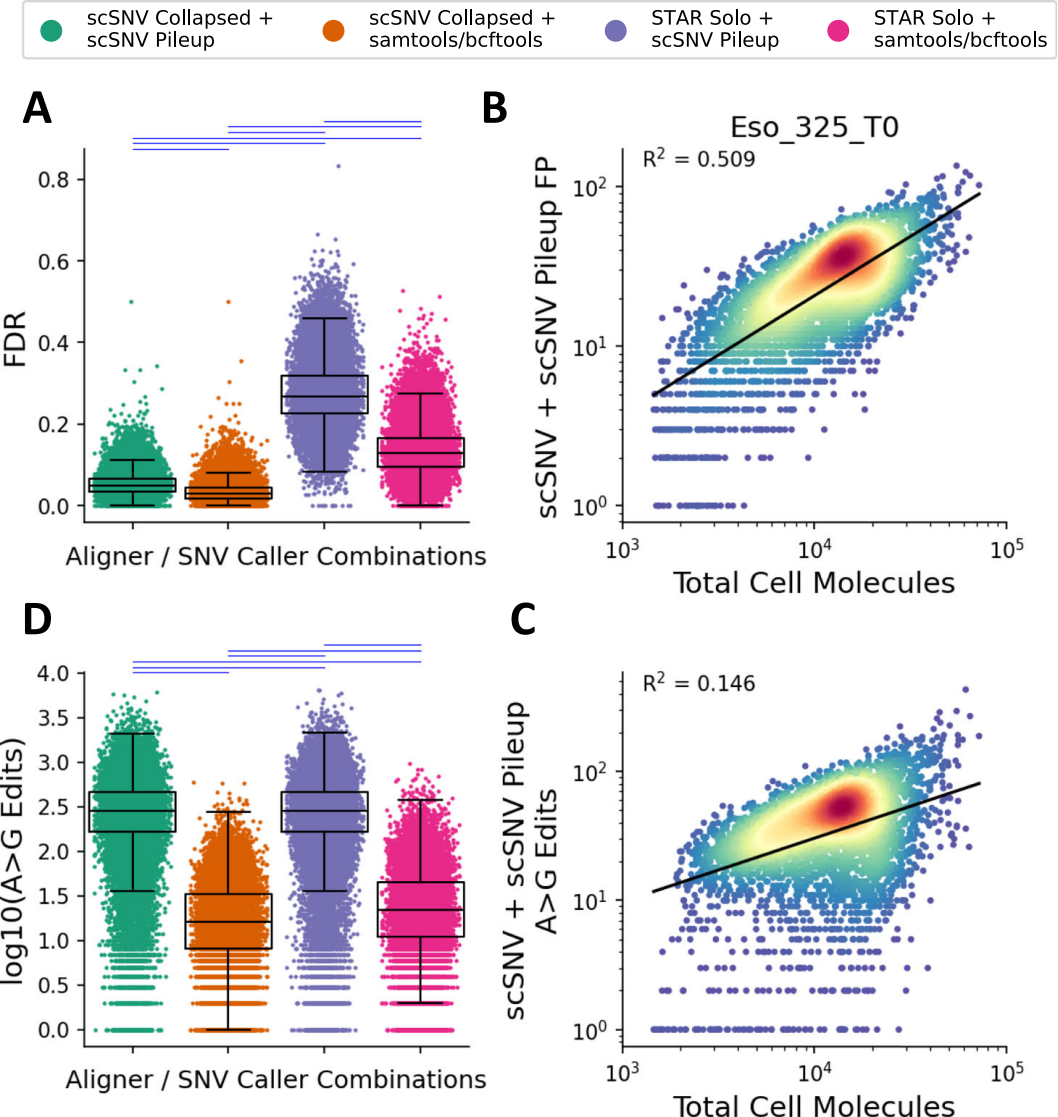
Assessing false-positive and A>G edit detection for single cells using the nine normal tissue samples (*N* = 36,581 cells) with matched WGS data. **a**, **c** The per-cell FDR (**a**) and log10(A>G Edit Count). Each aligner/SNV caller is indicated as per the legend, and the horizontal lines above the plot indicate the results of a two-sided Wilcoxon signed-rank test (*P* < 0.001 blue otherwise red). **b**, **d** Representative comparison of the number of false-positive SNVs (**b**) and A>G Edits (**d**) versus the number of spliced + unspliced molecules in the cells from Eso_325_T0, the *R*^2^ value and line of best fit as determined by ordinary least squares regression are indicated on the plot (*P*<0.001). The boxplots for **a** and **c** indicate the 25th, 50th, and 75th percentiles and the whiskers are 1.5 times the interquartile range

In our previous pseudo-bulk analyses, we demonstrated that alignments with STARsolo had an increased FDR (non-germline, non-A>G edit SNVs) compared to alignments with scSNV. Moreover, SNV calling with scSNV Pileup had a higher FDR than calling with samtools/bcftools regardless of the alignment method. Next, we sought to explore if these differences are reflected at the single-cell level. We calculated the per-cell FDR and FP rates using the pseudo-bulk samples and as expected our FDR results were consistent with our pseudo-bulk analyses (Fig. 5a). We observed that alignments with STARsolo had a much higher median FDR (0.268 with scSNV Pileup and 0.128 with samtools/bcftools) compared to alignments with scSNV (0.049 with scSNV Pileup and 0.029 with samtools/bcftools). In this case, each of the tool comparisons yielded a significant difference (*P* < 0.001, two-sided Wilcoxon signed-rank test) and a lower Pearson *R*^2^ correlation (~0.3–0.4 between alignment methods and ~0.8 using the same alignment methods, *P* <0.001). Moreover, we observed that samtools/bcftools generally had a lower FDR. Interestingly, we observed a low *R*^2^ (average 0.606 ± 0.091 across the nine samples *P* <0.001 ordinary least squares regression) when comparing the total false positives versus the spliced + unspliced molecules in each cell (Fig. 5b).

### SNV calling with scSNV Pileup detects more A>G edits per cell than samtools/bcftools

We sought to compare the A>G edit detection rate in single cells across the nine samples with matched WGS data in single cells. As we previously observed, SNV calling with scSNV Pileup yielded more A>G edit calls than with samtools/bcftools and similar rates between alignment methods. As expected, in single cells, we observed a much larger median number of A>G edits per cell for SNVs called with scSNV Pileup (281 with scSNV and 282 with STARsolo) compared to samtools/bcftools (16 with scSNV and 22 with samtools/bcftools). The differences between each of the methods were significant (*P* < 0.001, two-sided Wilcoxon signed-rank test) and the Pearson *R*^2^ correlation was high (>0.9, *P* < 0.001) using the same SNV caller and lower between SNV callers (~0.8, *P* < 0.001).

The results from the last two sections are consistent with our pseudo-bulk analyses where there was a trade-off between scSNV Pileup and samtools/bcftools in terms of detecting SNVs with lower minor allele fractions such as A>G edits and an increased false discovery rate.

### Collapsed molecules improve the identification of co-expressed SNVs

**Fig. 6:**
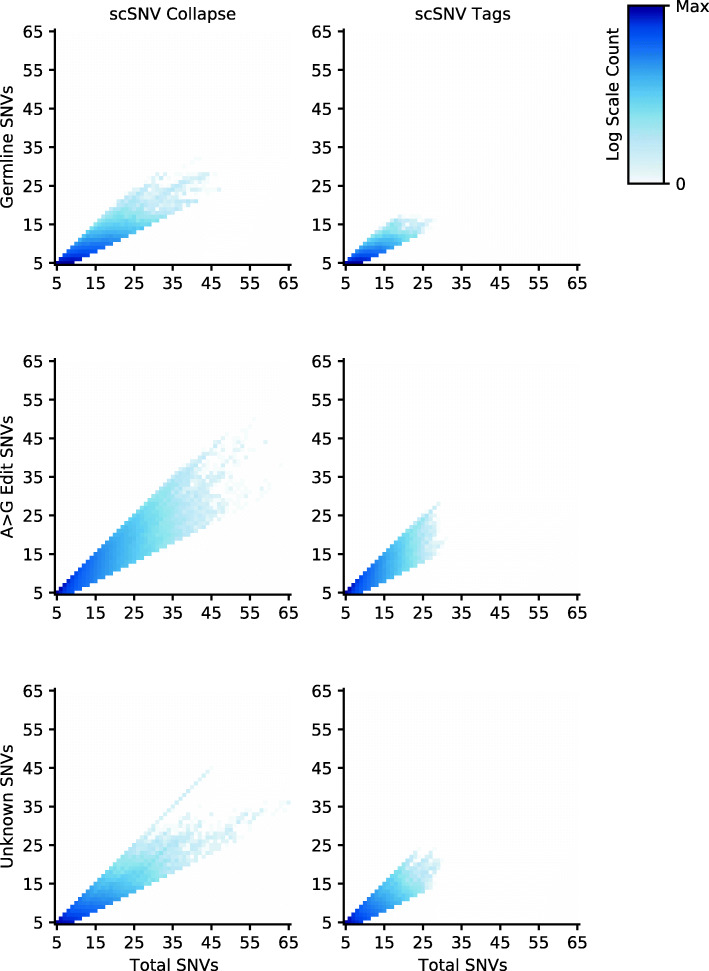
Comparing molecules (scSNV collapse) or tags (scSNV tags) with at least five co-expressed SNVs. For each sample, we identified the unique sets of SNVs detected by at least one molecule/read and we counted the number of germline SNVs, A>G edits, and unknown changes. For each set where the count of the maximal SNV type was more than half the total count, we assigned it as having a dominant SNV type. We then merged all of the sample sets together without removing duplicate entries. We calculated values for SNV data from scSNV (**a**, **c**, **e**) and Cell Ranger (**b**, **d**, **f**) and plotted 2D-histograms of dominant SNV type count versus the total number of SNVs in the molecule for SNVs overlapping germline (if available) and 1000 Genomes calls (**a**, **b**), A>G edits (**c**, **d**), and unknown SNVs (**e**, **f**). The log counts for each 2D bin are indicated by the color bars beside each heatmap

We further sought to explore the utility of collapsed molecules for the detection of SNVs co-expressed within the same molecule and the functionality of scSNV to efficiently extract per-cell SNV co-expression data. In this section, we defined an SNV as germline if it was in the matched DNA sequencing data (if available) or 1000 Genomes. For each sample, we examined SNV co-expression using SNVs called with scSNV Pileup as samtools/bcftools misses the majority of A>G edits. We only used alignments emitted by scSNV because and Cell Ranger and STAR are limited by default to 10 mismatches in a 96-bp read. As a comparison to demonstrate the increase in SNV co-expression detection, we compared the collapsed molecules from scSNV to the reads alignments without collapsing. We determined the unique sets of co-expressed SNVs for all twenty-two samples that had at least five co-expressed SNVs and a dominant SNV type, i.e., more than half of the SNVs (83.2 ± 4.34% of co-expressed SNV sets per sample). First, we observed that individual tag alignments are limited to detecting 31 SNVs, while collapsing identified up to 68 SNVs in a single molecule (Fig. 6). With scSNV, we could detect up to 32 germline SNVs compared to 17 for tags, 50 A>G edits compared to 28 for tags, and 45 unknown SNVs compared to 24 for tags. Next, we examined the purity of the dominant SNV type, i.e., the percentage of the SNVs in the set that were the dominant type, and found similar average purities for each SNV type between scSNV and Cell Ranger. For the germline SNV sets of 69.86 ± 13.40% for collapsing and 69.01 ± 12.58% for tags, for the A>G edit sets of 82.17 ± 15.32% for collapsing and 82.72 ± 15.33% for tags, and for the unknown SNVs of 73.31 ± 15.08% for collapsing and 76.12 ± 15.58% for tags. The high purity of A>G edits is expected as these are commonly found in regions of hyper-editing but may also indicate that using SNV co-expression may be a method to identify further edits that are co-expressed with known sites [24]. Finally, we observed a significant difference between the number of SNV’s detected per molecule (*P* < 0.001 two-sided Wilcoxon signed-rank test) for each comparison between scSNV with and without collapsing. Collectively, this demonstrates that collapsing molecular reads provides additional SNV co-expression information than examining SNVs using individual reads alone.

## Conclusion

Herein, we present scSNV, a novel pipeline to facilitate the accurate identification of SNVs and to explore their co-expression from dscRNA-seq data. One of the key innovations of scSNV is the collapsing of duplicate reads into consensus molecules we have called “collapsed molecules.” These collapsed molecules can be 400 bp or more in length with their size being limited to the size selection step during library generation. Furthermore, we demonstrated that the size distribution of these collapsed molecules is dependent on the sequencing saturation and PCR duplicate rate. A high saturation indicates that the sample has been sequenced to sufficient depth, while a high PCR duplicate rate may indicate problems with the complexity of the sample or that the sample was overamplified during the second library amplification step. Finally, we demonstrated that these collapsed molecules are useful to explore the co-expression of SNVs in single molecules. For example, we demonstrated that we can find regions of A>G hyper-editing with upwards of 50 edits in our samples. One limitation of our analyses is that we did not attempt to identify additional A>G edits by looking for A>G changes that were not identified as edits that are co-expressed with an annotated edit. This may be worth exploring in the future as a method to identify additional A>G RNA editing using co-expression data.

We have demonstrated that scSNV alignments with and without read collapsing have similar TPR and A>G identification rates as Cell Ranger and STARsolo, albeit a reduced FDR. The effect of collapsing on SNV calling is minor; however, collapsing reads negates count overinflation due to molecular and PCR duplicates. These results were consistent when using two different SNV calling methods, scSNV Pileup and samtools/bcftools. We explored the trade-off between the TPR, A>G edit calling rate, and FDR when using scSNV Pileup versus samtools/bcftools. The choice between the methods will depend on the user’s goal; for example, samtools/bcftools would be ideal if a user desires to minimize the FDR and is only interested in SNVs with a high allele fraction (>~0.20). However, if a user desires low allele fraction SNVs or A-to-G RNA edit calls, then scSNV Pileup would be more suitable despite the small increase to the FDR. We have demonstrated that a minor allele fraction cutoff of 0.25 reduced the FDR for scSNV Pileup calls with a minor reduction in TPR; however, when analyzing a sample consisting of 5 different pooled individuals, this cutoff would result in the loss of the majority of SNVs that are unique to each individual. In this case, minimal SNV filtering has been proven effective by two different genotype-based single-cell deconvolution methods and it may be worth exploring if alignments with scSNV with read collapsing offer an accuracy improvement compared to alignments with Cell Ranger [10, 11].

We evaluated a third SNV caller, Strelka2, which we found to have poor performance on pseudo-bulk dscRNA-seq data despite being recommended by a previous study [20]. However, the latter study focused on full-length plate-based scRNA-seq samples where each cell has higher coverage compared to dscRNA-seq tag-based methods and only examined simulated homozygous SNVs called individually on a random subset of the cells from a single dscRNA-seq library, which is different than the pseudo-bulk approach commonly used with dscRNA-seq data. Nonetheless, our simulated results with samtools/bcftools results were similar to their single dscRNA-seq library benchmarking, which suggests that Strelka2’s performance is not ideal for dscRNA-seq data samples. It should be noted that we did not evaluate the efficacy of calling SNVs by treating each individual cell from dscRNA-seq as a bulk sample, as this would be computationally challenging to manage (generating and calling SNVs on thousands to tens of thousands of alignment files) and unlikely to increase the true-positive rate and may increase the false discovery rate. Nonetheless, a modified version of this approach may increase the sensitivity of SNV callers that do not detect rare SNVs by default such as samtools/bcftools, where multiple subsets of the bulk sample (such as cell clusters) could be extracted and SNVs called on their aggregated alignments. This approach would be less useful for our scSNV Pileup method where a low allele fraction is used by default for our pileup, which can be revised later depending on the sample, for example, using the 0.25 cutoff we found to be effective on our benchmarked samples.

We also demonstrated that the sparsity of dscRNA-seq data led to each cell only expressing a small subset of the total true-positive SNVs detected in a pseudo-bulk sample and the low coverage of heterozygous SNVs in many of the cells leads to a reduced TPR for individual cells. To further validate that the per-cell reduction in TPR was due to the aforementioned heterozygous SNVs, we demonstrated that the per-cell TPR reaches an average of one as we selected for homozygous SNVs by increasing the average minor allele fraction cutoffs applied to the SNV calls within each cell. Furthermore, simulation of 25,000 homozygous SNVs in three of the biological samples also yielded a high TPR (>0.94), which is consistent with our matched WGS benchmarking approach when our analyses were limited to homozygous SNVs or SNVs with high coverage.

Collectively, our benchmarking of dscRNA-seq alignment and SNV calling methods validates that true-positive germline (and somatic) SNVs can be accurately called from dscRNA-seq pseudo-bulk samples and quantified in single cells. The reduction in false-positive SNV calls using scSNV compared to Cell Ranger and STARsolo will also be desirable when looking for subclonal SNVs. Furthermore, the sparsity of dscRNA-seq data limits the accurate quantification of heterozygous SNVs in single cells from regions of low coverage.

The compute time for STARsolo was much lower compared to scSNV and Cell Ranger, with similar TPR rates, which would make STARsolo a desirable option if a user is only interested in quantifying a known set of germline or somatic SNVs. In the future, it may be worth integrating some of our alignment processing steps to improve the FDR for STARsolo leading to a rapid method for SNV identification in dscRNA-seq data. It would also be worth exploring if our read collapsing strategy could be applied to STARsolo alignments; however, some pre-processing of the alignments may be necessary to avoid issues where the mRNA tag alignments from a given molecule are not compatible, for example, if a portion of the reads have a false-positive novel splice junction instead of an insertion or deletion. Finally, the properties of false-positive SNVs from each of the alignment methods could be explored to develop a classifier to better identify false-positive SNVs from collapsed molecules.

In the future, SNV co-expression and tag collapsing may improve the accuracy of demultiplexing dscRNA-seq samples [2] since germline SNVs unique to each pooled genotype should not be co-expressed within the same collapsed molecule. Moreover, co-expression data may be useful when integrating dscRNA-seq with matched single-cell DNA copy number data where both datasets are sparse and therefore maximizing SNV information to infer subclones is essential. Finally, the idea of collapsing SNVs could be implemented using tag assembly from their sequences rather than their alignments. The mRNA tags could be grouped by their cell barcode and UMI and followed by de novo assembly. The assembled longer reads could be mapped to the genome/transcriptome, which could facilitate the identification of large insertions, deletions, and gene fusions. However, this may be technically challenging to implement as the tags from a UMI duplicate may not overlap but should still cover ~100–500 bp of transcript sequence depending on the number of UMI duplicates in the molecule.

In conclusion, scSNV represents an accurate dscRNA-seq pipeline that minimizes SNV false-positive calls and facilitates the analysis of SNV co-expression at the cellular and molecular levels.

## Methods

### Genome build information

GRCh38 human genome annotations from Ensembl build 94 [25] were downloaded and used for all of the analyses. 1000 Genomes [23] phase 3 common SNPs were downloaded from Ensembl, and mitochondrial mutations were downloaded from the 1000 Genomes site. The two VCF files were merged and filtered with bcftools to select bi-allelic variants with a frequency greater than 0.01. Known RNA edits were downloaded from REDIportal [26].

### Previously published samples

Three T0 sets of dscRNA-seq samples from lung, spleen, and esophagus samples with matched whole genome sequencing were retrieved from the HCA data access portal [18, 27] (see Additional file 1: Table S1). The PBMC 4k and T-cell samples were retrieved from the 10X Genomics website. The five pancreatic ductal adenocarcinoma samples were downloaded from the genome sequencing archive (accession CRP000653) [19].

### Processing of whole genome samples

Paired-end reads for each sample were individually mapped with BWA-mem v0.7.17-r1188 [16], sorted with samtools [28] v1.9, and had duplicates marked with picard 2.18.20-1 (http://broadinstitute.github.io/picard). Bi-allelic SNVs were called with Strelka2 [12] with the default options.

### Overview of scSNV

scSNV consists of 5 alignment steps: (1) index generation (only needed once), (2) barcode counting, (3) QA and read mapping, (4) UMI collapsing, and (5) collapsed BAM file generation. Additionally, the code to generate simulated mixture data and the auxiliary scripts used to process and annotate the data are included with the package.

### Full-length transcript index construction

scSNV generates a full-length transcript index from a GTF and genome fasta file. By default, transcripts annotated as retained_intron, nonsense_mediated_decay, non_stop_decay, and transcripts with a length less than 100 bp are not included. The transcript fasta file is indexed using BWA-mem [16] with default parameters.

### Read QA, mapping, and barcode correction

We have adopted a similar cell barcode counting and correction scheme as Cell Ranger [5]. In brief, all of the cell barcodes that match a known barcode are counted. During the mapping step, if a barcode does not match a known barcode but is one mismatch away from one or more known barcode(s), it is assigned to the known barcode with the highest count.

To remove low-quality reads prior to alignment, we apply the following QA steps: (1) The cell barcode must match a known barcode after correction and must not contain any ambiguous bases. (2) The UMI must not contain any ambiguous bases. (3) Poly(A) sequences are trimmed off of the read tag ends and the sequence remaining after trimming must be at least half the reads length; otherwise, the read is discarded. (4) An optional DUST score [29] is calculated for the remaining read tag and reads with a DUST score greater than a user-defined threshold are discarded. Reads that pass all of the QA steps are processed for alignment tag mapping.

The alignment tag for each read is mapped independently to a full genome index and full-length transcript index using BWA-mem [16] C API with the default alignment scoring options. Each transcript alignment is projected to genomic coordinates. A minimum BWA-mem alignment score cutoffs of 45 is used. To mitigate a common source of false-positive mismatches, alignments that pass the scoring threshold are trimmed back if they overhang a splice site by less than or equal to 5 base pairs [15]. To identify multi-mapped reads, a minimum alignment score of |maximum alignment score - 3| is calculated. All alignments with scores greater than or equal to the minimum alignment score are retained. Each alignment from a single read tag is then given an alignment type based on the following schema:

Unmapped: no alignments present
Antisense: the read is derived from the antisense strand of an annotated gene
3) Exonic: all full-length transcript alignments and genomic alignments with more than 25 base pairs of the read overlapping an annotated exon from a single gene
Intronic: more than 5 base pairs of the read overlap an annotated intron (i.e., no overlap with an exon) from a single gene
Intergenic: a genomic alignment that is not annotated as antisense, exonic, ambiguous, or intronic
Ambiguous: genomic alignments where the read overlaps more than one gene

Finally, the set of alignments for a given tag are annotated as follows:

1. Multi-mapped: the read maps to more than one location, for example, two different genes or one gene and an intergenic region
2. Antisense: all of the reads alignments are antisense to a gene
3. Ambiguous: all of the reads alignments are ambiguous or the read has both an intronic and exonic alignment within the same gene
4. Exonic (spliced): all of the reads alignments map to exonic regions of the same gene
5. Intronic (unspliced): there is a unique intronic alignment to a single gene

Only intronic and exonic alignments are used for UMI collapsing and quantification. Finally, this step emits position-sorted BAM files for reads that map to exonic and intronic regions that can be merged after UMI deduplication to produce deduplicated and collapsed BAM files (see below).

### Spliced and unspliced alignment collapsing and counting

A UMI group is defined as a set of reads with a matching gene ID, cell barcode, and UMI; these reads are expected to be derived from the same molecule. It is worth noting that these reads do not have to have the same mapping position to be considered duplicates. To extend groups to handle mismatches within the UMI, we apply the directionality collapsing algorithm [30] to all of the UMIs from the same gene ID and barcode. A UMI group is annotated as unspliced/intronic if at least one of the reads in the group was annotated as an intronic alignment the read is annotated as spliced/exonic otherwise. We have extended the UMI collapsing algorithm to count PCR duplicates, which are read alignments in a UMI group that have the same mapping position. Finally, after UMI collapsing, we implement a method similar to Cell Ranger, where the same corrected UMIs from a given barcode are grouped together, and if the same UMI maps to multiple genes, the one with the highest supporting reads is retained and the others are discarded as these reads are likely to represent mapping errors.

For each cellular barcode, we output a list of alignment statistics including the number of alignments from each alignment type defined in the mapping step, the number of unique exonic and intronic molecules, the PCR duplicate rate, the UMI duplicate rate, and an option to count the number of unique molecules from user-defined lists of genes, for example, mitochondrial genes. The molecule count data, alignment statistics, and other information are written to an HDF5 (http://www.hdfgroup.org/HDF5/) file to permit easy access to the data in any programming language that has an HDF5 library.

After the UMIs are deduplicated, the sorted BAM file(s) are merged and deduplicated where PCR duplicates and UMI duplicates are annotated. Reads from the same UMI group are then collapsed to generate a BAM file where all of the reads in a UMI group are merged into a consensus sequence. For each aligned base that has more than one overlapping read, we determine a consensus base where at least 60% of the reads have the same base; otherwise, the consensus base is set to ambiguous (*N*). We set the quality of the base to the maximum base quality supporting the consensus base. For insertions and deletions (indels), we require at least 60% of the reads overlapping the indels position to have the exact same sequence.

One minor limitation of our implementation is that if all of the UMI groups do not map linearly across the genome, the group is not collapsed. In our experience, this happens to a small fraction (typically <0.01%) of the reads.

### Identifying cells

First, we only examine cell barcodes with at least one spliced molecule and we determine a rough estimate of the number of cells using the “elbow” approach previously described. Next, while exploring the data, we observed that samples tend to have outlier barcodes with high proportions of mitochondrial (MT) molecules, low sequencing saturation or PCR duplicate rates, or a high proportion of reads that were barcode corrected (see the “Methods” section above). In each of these cases, we applied a median absolute deviation (MAD) filter with the following parameters:

1. For MT DNA percent, we used the median + min(5 × MAD, 20) as a maximum cutoffs
2. For saturation and PCR duplicates, we used the median − min(3 × MAD, 10) as the minimum cutoffs
3. For the percent of barcode corrected reads, we used the median + max(10 × MAD, 1.5) as the maximum cutoffs

We include an example script that implements our cell determination algorithm that can output the data as a python hdf5 file and anndata [31] file. The latter can be used with tools such as scanpy [31] and Seurat [32].

### Benchmarking scRNA-seq alignment methods

All alignments were run on a custom genome build using Ensembl 94. In brief, STARsolo from STAR package v2.7.5c [14] and Cell Ranger v2.1.1 and scSNV were limited to at most 24 threads. Cell Ranger was run with the --nosecondary flag to disable secondary analysis as this increases the runtime calculations for Cell Ranger. STARsolo was indexed without pre-filtering the genome annotations, which is a slight deviation from Cell Ranger and run with the following arguments:

*--limitBAMsortRAM 41995186164 --soloType CB_UMI_Simple --soloCBwhitelist 737K-august-2016.txt --soloUMIfiltering MultiGeneUMI -soloUMIfiltering MultiGeneUMI --soloCBmatchWLtype 1MM_multi_pseudocounts --outSAMattributes NH HI nM AS CR UR CB UB GX GN --outSAMtype BAM SortedByCoordinate*

The 737K-august-2016.txt is the white list of barcodes for V2 dscRNA-seq libraries provided by 10X Genomics and is mirrored in the scSNV github repository.

To quantify unspliced molecular counts for a subset of samples between scSNV and Cell Ranger, the velocyto [33] python package v0.17.17 was used without repeat filtering. The filtered output matrices from Cell Ranger were rederived using the raw output matrices and the barcodes identified using scSNV to simplify comparisons with scSNV.

For reproducibility, we have mirrored the source code for Cell Ranger v2.1.1 at:

https://github.com/GWW/cellranger_211_mirror.

### SNV calling from single-cell data

We developed a pileup tool to take into account the strand-specific nature of 10X Single Cell libraries and to quantify the number of supporting barcodes, molecule counts for each strand, and the maximum distance from SNV to the closest read 5′- or 3′-end. For collapsed molecules, we extend the idea of a mismatch being near a read-end to include internal read-ends. For example, a collapsed molecule could consist of three reads that do not overlap and the reads-ends internal to the read could have false-positive SNVs at one of its ends due to a mis-spliced read. The program works on both the collapsed and mRNA tag-aligned BAM files to facilitate comparison with Cell Ranger. We set default filtering conditions for SNV sites to only count reads at a given position if it has a minimum base quality of 20 and for the read to be derived from. For Cell Ranger and STARsolo, we implemented additional read filtering to select for reads that were mapped uniquely (mapping quality 255, tags: MM:1, RE: E/N) to intronic (mapping to a single gene) or exonic regions. It should be noted that for Cell Ranger we did not remove reads that were marked as duplicates as we observed that this substantially reduced the number of SNVs identified by Cell Ranger.

We chose our filtering parameters to maximize the detection of mutations that may be important to genotype rare populations. For example, filtering by allele fractions may cause us to filter out mutations that may be informative for genotyping. Instead, we require that each potential SNV be covered by at least 5 unique cell barcodes, at least 2 unique barcodes supporting the alternative allele, a minor allele fraction of at least 0.01, and have at least one read with a supporting mismatch outside of the first and last 5 base pairs of the read. All of the data is emitted from scSNV’s pileup command into a HDF5 file for easy loading into a programming language that has an HDF5 library.

To call SNVs with samtools/bcftools [13], we used the following flags with Cell Ranger and STARsolo to process all reads including PCR duplicates as excluding these caused a huge false-negative rate: --ff 2820 -Q 30 -A -x and for scSNV we used the following: -Q 30 -A -x. To call SNVs with Strelka2, we used the default options but included the --rna tag to enable RNA-seq filtering parameters.

### Annotating SNVs from single-cell RNA-seq data

We developed a custom python script to annotate each SNV (available in the scSNV code repository). First, we refined the list of passed SNVs by requiring that 95% of the reads supporting the SNV consist of the reference and alternative allele, and since 10X single-cell libraries are strand-specific, we inferred the strand for each potential SNV by requiring that at least 90% of the reads covering the SNV be derived from the same strand; otherwise, the strand was marked as unknown. This filtering step tends to retain ~98% of the SNVs. We annotated each SNV with overlap to RepeatMasker annotations downloaded from UCSC GRCh38 [34] and known A-to-I RNA edits from REDIportal [26] (both retrieved on November 22, 2019). The REDIportal annotations were transformed from hg19 to GRCh38 using UCSC’s liftOver [35] tool. An SNV was considered a potential A-to-I RNA edit if it was an A-to-G change, found in REDIportal, or overlapped an Alu element. Each SNV was further annotated if it matched a SNV found matched whole genome sequencing data (if available) and/or if it was found in 1000 Genomes common SNPs.

### Benchmarking SNV calling using matched genome sequencing data

We only used SNVs called as bi-allelic and strand-specific that were consistent across every method that called the SNV, i.e., the same alternative allele and inferred strand. We limited our analyses to regions that had at least 1X coverage with a base quality ≥ 20 by one of the alignment methods in a given dscRNA-seq sample and at least 15X coverage from reads with a mapping quality ≥ 20 and base quality ≥ 20 in the matched exome or WGS data. We called an SNV as identified in the matched DNA sequencing data if there were and 2 alternative bases with a mapping quality ≥ 20 and base quality ≥ 20. This includes SNVs that were not called by Strelka2 in the genome data as we wanted to be sure there was no evidence of an SNV at that position. We identified SNVs as true positives if they were called in the DNA sequencing data and the dscRNA-seq data as false positives if they were covered in the genome sequencing data without a call but called in the dscRNA-seq data, and false negatives if they were called in the genome data and had at least 5 barcodes covering the position with base quality ≥20.

### Simulation homozygous SNVs in biological dscRNA-seq samples

We simulated 25,000 homozygous SNVs for each of three different samples (Eso_325_T0, Lung_367_T0, and Spleen_325_T0), by randomly substituting positions in the reference genome with the following constraints: (1) the SNV must be at least 10 bp away from a SNV call from scSNV or STARsolo alignments using scSNV Pileup or samtools/bcftools and (2) at least 5X coverage in one of the samples (to avoid simulating the majority of SNVs in regions with 1X coverage). The modified genome was indexed and the reads were mapped with scSNV and STARsolo and SNVs called using scSNV Pileup and samtools/bcftools as described in the previous section. We classified a SNV as a true positive if there was at least one alternative allele expressed in a given SNV/cell pair and a false negative if the loci had no alternative allele expression but had detectable coverage in the modified genome alignment or the original unmodified genome. The latter constraint was included as alignments to the modified genome may affect the coverage of a given SNV in a particular cell, for example, if the SNV was near a read-end and the alignment tool soft clipped the read instead of emitting a mismatch.

### Esophageal adenocarcinoma sample collection and organoid growth

Patient blood samples were used for germline exome calls. Similarly, 2 pieces of endoscopic biopsy or resected tumor were snap frozen and stored at −80°C. The samples were thawed and used for DNA extraction with the Qiagen DNeasy Blood and Tissue Kit (Qiagen Inc, Venlo, NL). The somatic and germline exomes for EAC_46 and EAC_92 were sequenced as part of another study that is currently under revisions. Finally, all methods were performed in accordance with relevant guidelines and regulations. Exome sequencing data and organoid growth protocols were previously published by Derouet et al. [36].

### Preparation of single-cell suspension from EAC organoids and scRNA-seq

Organoid samples were dissociated using TrypLE for 10 min at 37°C. The melted suspension was then transferred to a non-adherent 6-well plate and visualized using a bright field microscope. Single organoids were then picked using a pipet and transferred to a 96-well plate. Two hundred microliters of TrypLE was added to each well containing a single organoid and incubated for 20 min at 37°C. Then, 200μL of cold fresh Advanced DMEM/ F12 medium was added and then the plate was spun down at 4°C. The 96-well plate was then placed on ice and each pellet was resuspended in 100 μL cold fresh Advanced DMEM/F12 containing 10 μM of ROCK inhibitor (R&D Systems, USA). The cell suspension was then filtered through a 40-μm cell strainer and stored on ice. Samples were processed using the 10X genomics Chromium V2 3′-dscRNA-seq kit as per the manufacturer’s instructions using an input of 5000 cells. The 10X libraries were sequenced using an Illumina HiSeq 2500 or NextSeq sequencing machine following the manufacturer’s directions.

## Supplementary Information

**Additional file 1: Supplementary tables**.

**Additional file 2: Supplementary figures**.

**Additional file 3.** Review history.

## Data Availability

scSNV is open source, multithreaded and implemented in C++ and available under the MIT license on GitHub at https://github.com/GWW/scsnv [37]. The exact version of the source code used in this manuscript is available at 10.5281/zenodo.4458260 [38]. Seventeen of the samples analyzed in this study are publicly available. Nine with matched WGS data through the Human Cell Atlas Data Coordination Platform and NCBI BIOPROJECT access code PRJEB31843 [18], three from the 10X genomics website [39], and five from the Genome Sequence Archive project PRJCA001063 [19] (see Additional file 1: Table S1 for specific sample IDs). The five esophageal adenocarcinoma organoid dscRNA-seq samples generated for this analysis and previously published matched exome sequencing data are available through EGA at EGAD00001007525 [40].
