# Supplementary material for: scSNV: accurate dscRNA-seq SNV co-expression analysis using duplicate tag collapsing
Source: Genome Biol. 2021 May 7;22:144. doi: 10.1186/s13059-021-02364-5 (PMC8103760; doi:10.1186/s13059-021-02364-5)
Supplement: Supplementary file 2 — Additional file 2: Supplementary figures. [file 13059_2021_2364_MOESM2_ESM.pdf]

Additional File 1 – Supplemental Figures and Legends

Figure S1. Run time comparison of scSNV, STAR Solo and Cell Ranger across 22 dscRNA-seq samples colored by the sample series. The runtime for scSNV (circle), Cell Ranger (triangle), and STAR Solo (square) in minutes versus the total number of reads in millions. The black lines indicate the ordinary least squares regression fit for scSNV (solid), Cell Ranger (dashed), and STAR Solo (dotted). For scSNV: $R^2 = 0.466$, $-\log_{10}(P) = 3.336$, Cell Ranger: $R^2 = 0.233$ $-\log_{10}(P) = 1.638$, and STAR Solo $R^2 = 0.033$, $-\log_{10}(P) = 0.381$.

Figure S2. Comparison of spliced (**A, B, C**) and unspliced (**D, E, F**) counts from scSNV and Cell Ranger / Velocyto. For each plot we are comparing the same set of barcodes extracted from scSNV and Cell Ranger (CR) using the quality barcodes identified by scSNV. (**A, D**) the total number of spliced and unspliced molecules for each barcode for scSNV (y-axis) and Cell Ranger (x-axis). The black diagonal line shows where values would be equal. (**B, E**) For each barcode pair from scSNV and Cell Ranger we calculated the spearman correlation between the spliced and unspliced counts between scSNV and Cell Ranger / Velocyto. (**B, E**) boxplots of the spliced and unspliced counts for each sample. The x-axis includes the same name and number of cells compared. (**C, F**) the Spearman correlation for all samples vs the total molecules identified by scSNV for each barcode. Samples are colored by their tissue type as per the legend.

Figure S3. Collapsed read lengths for molecules with (**A**) two reads, (**B**) three reads, (**C**) four reads, and (**D**) five or more reads. Samples are ordered by their sequencing saturation (lowest to highest). For each sample the length distribution of the number of collapsed bases for each read was down-sampled to 20,000 reads, smoothed using gaussian kernel density estimation, and then scaled so the minimum and maximum value ranged from 0 to 1. The density values are indicated by the color bar. The sequencing saturation and PCR duplicate rate are indicated by the 2nd and

third color bars while the tissue type for each sample is indicated by the top color as per the legends.

Figure S4. SNV calling accuracy using matched whole genome data to estimate F1-scores (top), TPRs (middle) and FDRs (bottom) on the y-axis stratified by increasing minor allele fraction cut-offs (x-axis) for four alignment methods as indicated by the line color and three SNV calling tools as indicated above each graph. The median line for each tool and method is indicated with a solid line, samples with matched exome data are indicated with a dotted line and samples with WGS data are indicated with a dashed line.

Figure S5. Determining a minor allele fraction cutoff that maximizes the TPR and minimizes the FDR for SNVs called using scSNV Pileup. The TPR is on the x-axis and FDR on the y-axis and the lines are from applying increasingly stringent allele fraction cut-offs from 0.01 to 1.00. Each of the eleven samples with matched WGS data are shown individually as indicated above the plot, the color of each line indicates the alignment tool and the dot indicates the algorithmically determined elbow. The minor allele fraction cutoff is indicated within each plot.

Figure S6. The number of A>G edits detected at various minor allele fraction cutoffs for the eleven samples with matched DNA data and constrained to regions of high quality using the DNA data. The three SNV calling methods are indicated above the plot and the alignment methods are indicated by the color of the sample. The median is the solid line, the samples with matched exome data are dotted and with matched WGS are solid.

Figure S7. Heatmaps of the the average TPR (first column) and percent of the true positives detected in each cell (second column) across the cells from all nine samples after applying increasing coverage cutoffs (y-axis) and average minor allele fraction cutoffs across the cells from

each sample (x-axis) for alignments with scSNV and SNV calls using samtools/bcftools (A), STARSolo with scSNV Pileup (B), and STAR Solo with samtools/bcftools (C).

Figure S8. Assessing the effect of saturation on SNV coverage overinflation when using tag counts rather than collapsed molecule counts. For demonstration purposes we used SNVs called with scSNV Pileup for each of the comparisons. A) The average log2 fold change in STAR Solo tags versus scSNV collapsed molecule counts for true positive SNVs detected in each of the nine samples with matched WGS data. The error bars indicate the standard deviation for each sample. B-D) Examples of the STAR Solo tag coverage versus scSNV collapsed molecule coverage for samples with various saturation percentages as indicated above the plots.

Supplemental Table S1. Samples processed in this study

Supplemental Table S2. Sample SNV summary data for accuracy calculations

Supplemental Table S3. Summary of accuracy rates for individual cells

Figure S1

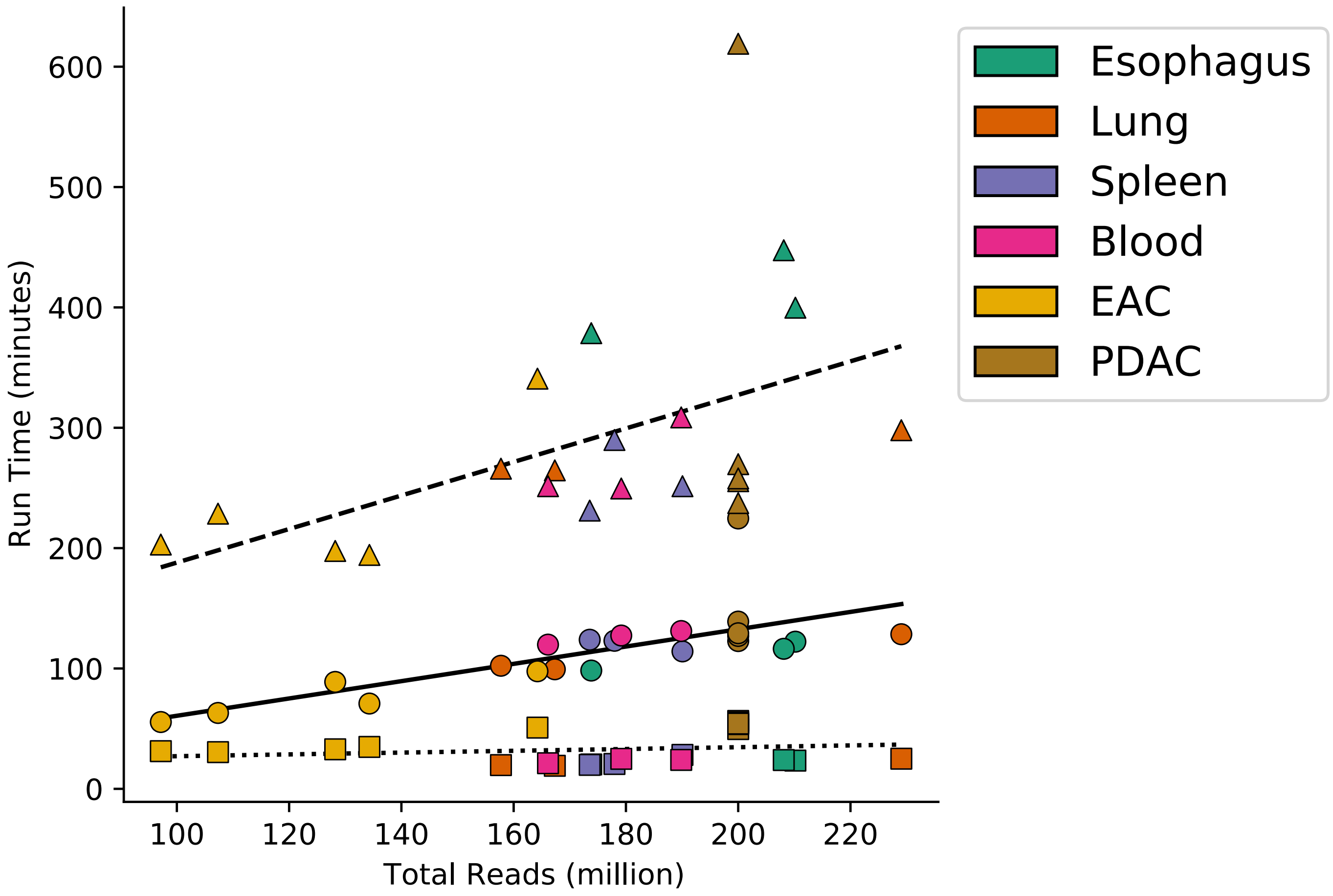
Run Time (minutes)
600
500
400
300
200
100
0
100
120
140
160
180
200
220
Total Reads (million)
Esophagus
Lung
Spleen
Blood
EAC
PDAC

Figure S2

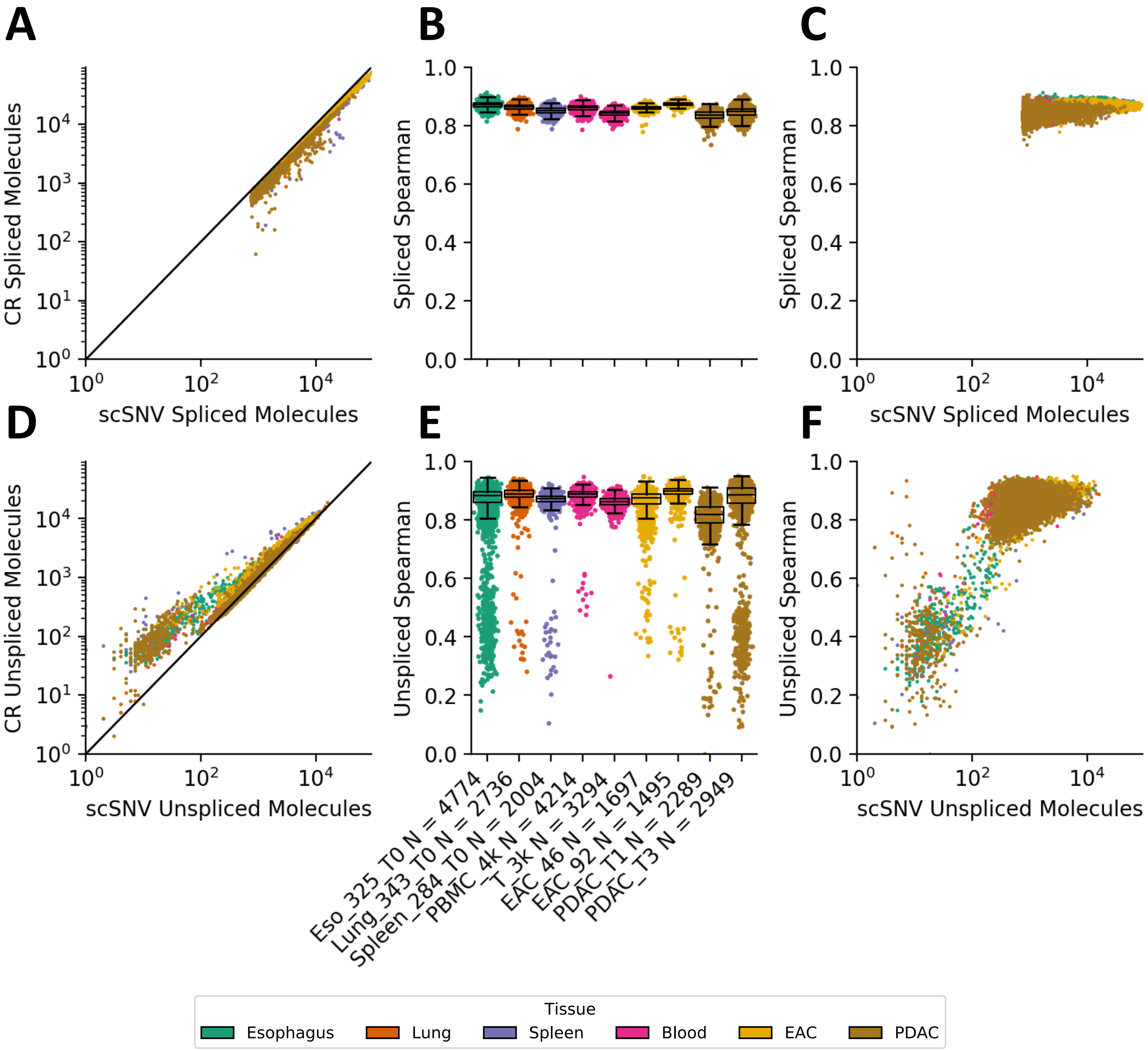
A
B
C
D
E
F
CR Spliced Molecules
scSNV Spliced Molecules
Spliced Spearman
CR Unspliced Molecules
scSNV Unspliced Molecules
Unspliced Spearman
Eso_325_T0 N = 4774
Lung_343_T0 N = 2736
Spleen_284_T0 N = 2004
PBMC_4k N = 4214
T_3k N = 3294
EAC 46 N = 1697
EAC 92 N = 1495
PDAC_T1 N = 2289
PDAC_T3 N = 2949
Tissue
Esophagus
Lung
Spleen
Blood
EAC
PDAC

Figure S3

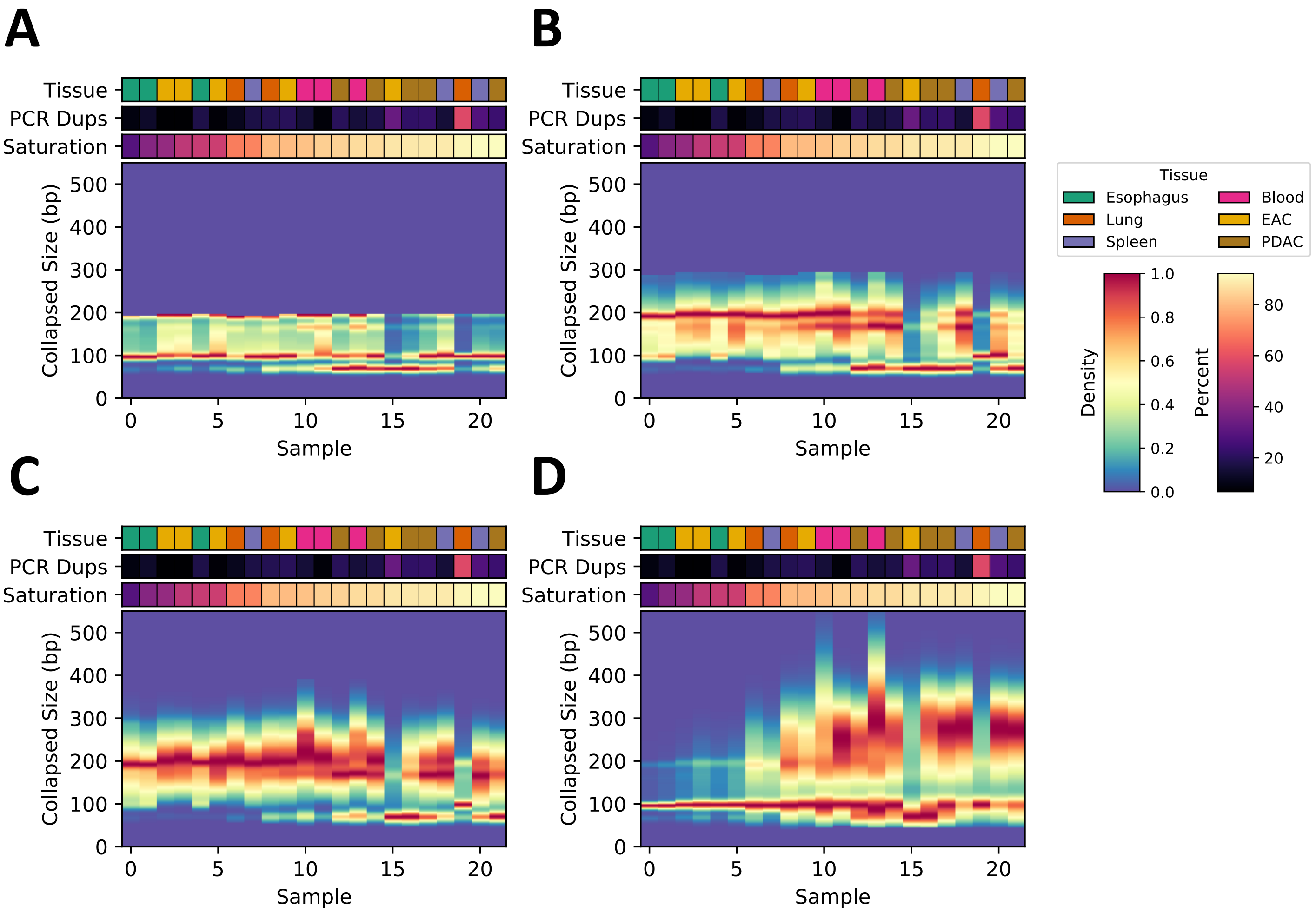
A
B
C
D
Tissue
PCR Dups
Saturation
Collapsed Size (bp)
Sample
0
100
200
300
400
500
0
5
10
15
20
Tissue
Esophagus
Lung
Spleen
Blood
EAC
PDAC
Density
Percent
0.0
0.2
0.4
0.6
0.8
1.0
20
40
60
80

Figure S4

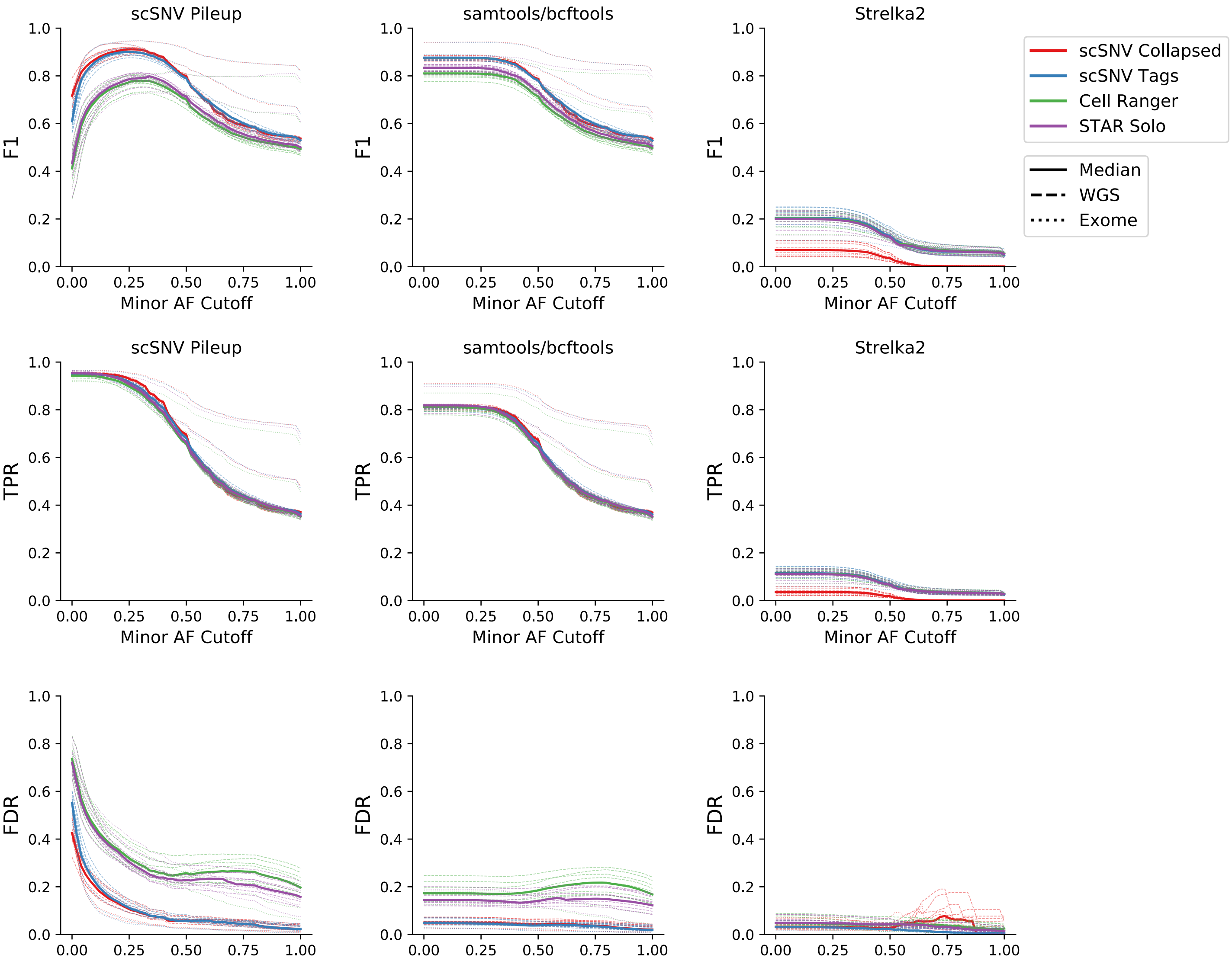
scSNV Pileup
samtools/bcftools
Strelka2
F1
TPR
FDR
Minor AF Cutoff
0.0
0.2
0.4
0.6
0.8
1.0
0.00
0.25
0.50
0.75
1.00
scSNV Collapsed
scSNV Tags
Cell Ranger
STAR Solo
Median
WGS
Exome

Figure S5

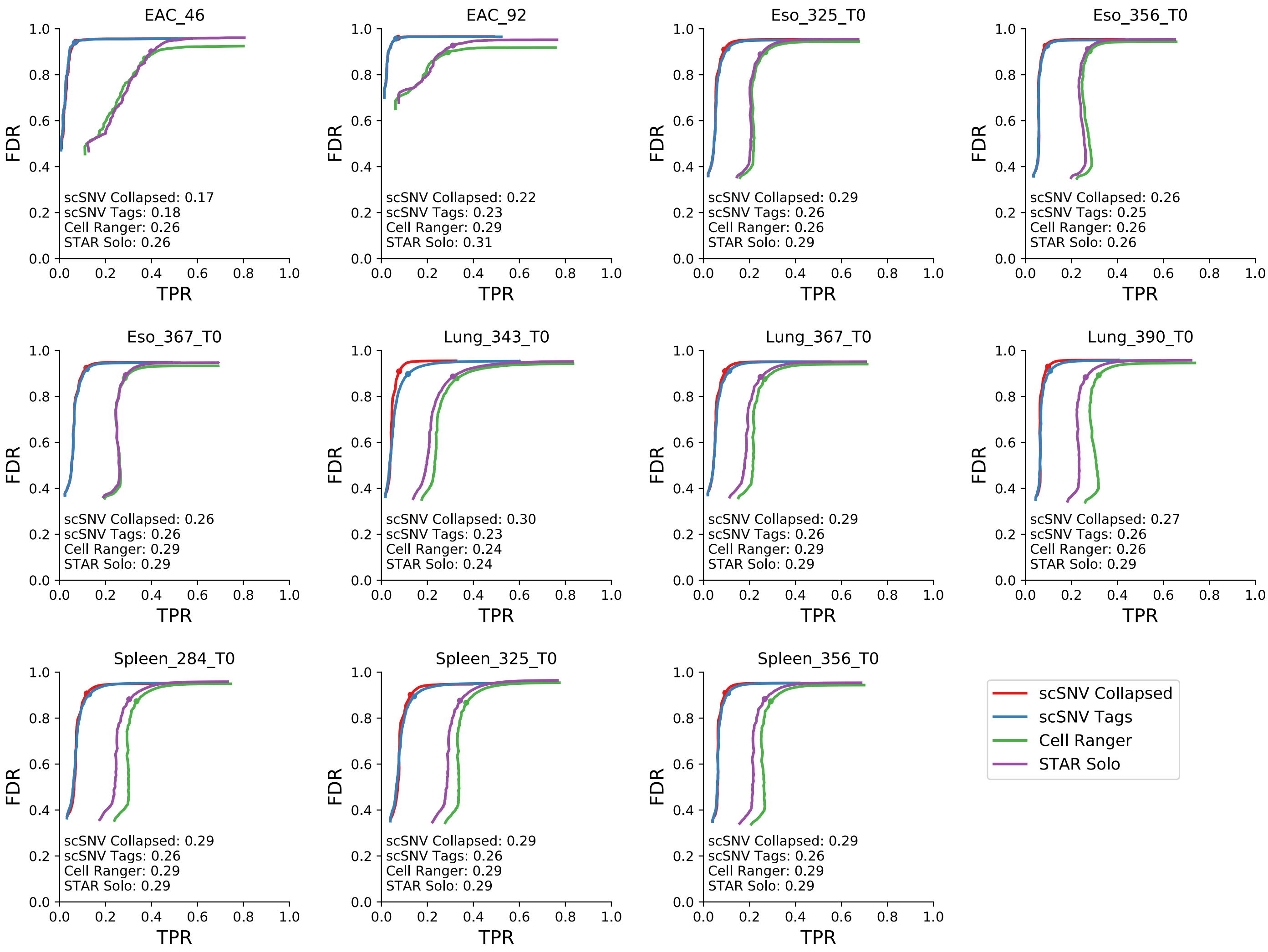
EAC_46
scSNV Collapsed: 0.17
scSNV Tags: 0.18
Cell Ranger: 0.26
STAR Solo: 0.26
EAC_92
scSNV Collapsed: 0.22
scSNV Tags: 0.23
Cell Ranger: 0.29
STAR Solo: 0.31
Eso_325_T0
scSNV Collapsed: 0.29
scSNV Tags: 0.26
Cell Ranger: 0.26
STAR Solo: 0.29
Eso_356_T0
scSNV Collapsed: 0.26
scSNV Tags: 0.25
Cell Ranger: 0.26
STAR Solo: 0.26
Eso_367_T0
scSNV Collapsed: 0.26
scSNV Tags: 0.26
Cell Ranger: 0.29
STAR Solo: 0.29
Lung_343_T0
scSNV Collapsed: 0.30
scSNV Tags: 0.23
Cell Ranger: 0.24
STAR Solo: 0.24
Lung_367_T0
scSNV Collapsed: 0.29
scSNV Tags: 0.26
Cell Ranger: 0.29
STAR Solo: 0.29
Lung_390_T0
scSNV Collapsed: 0.27
scSNV Tags: 0.26
Cell Ranger: 0.26
STAR Solo: 0.29
Spleen_284_T0
scSNV Collapsed: 0.29
scSNV Tags: 0.26
Cell Ranger: 0.29
STAR Solo: 0.29
Spleen_325_T0
scSNV Collapsed: 0.29
scSNV Tags: 0.26
Cell Ranger: 0.29
STAR Solo: 0.29
Spleen_356_T0
scSNV Collapsed: 0.29
scSNV Tags: 0.26
Cell Ranger: 0.29
STAR Solo: 0.29
FDR
TPR
scSNV Collapsed
scSNV Tags
Cell Ranger
STAR Solo

Figure S6

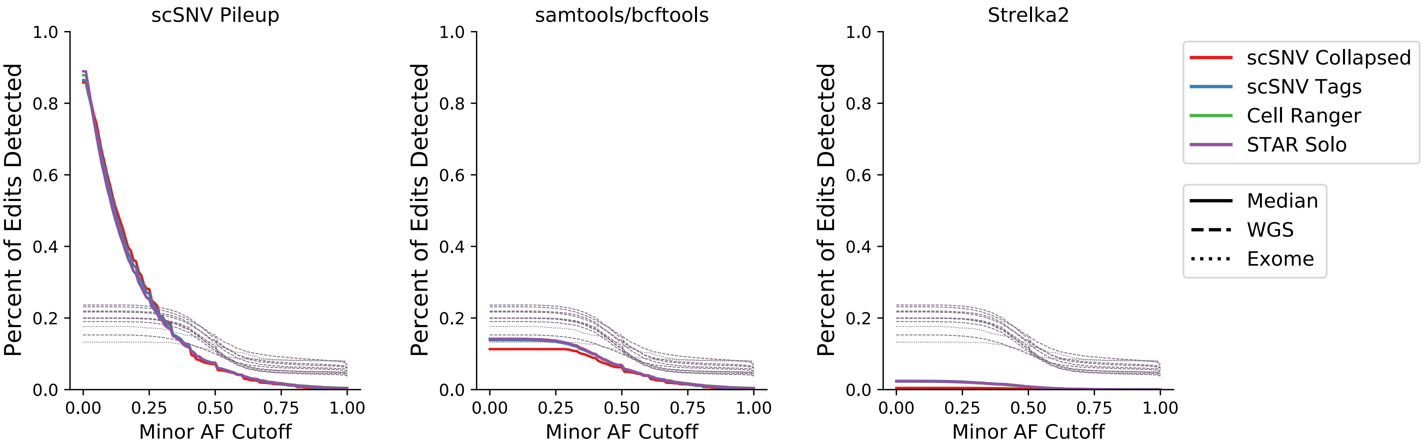

scSNV Pileup
samtools/bcftools
Strelka2
Percent of Edits Detected
Minor AF Cutoff
1.0
0.8
0.6
0.4
0.2
0.0
0.00
0.25
0.50
0.75
1.00
scSNV Collapsed
scSNV Tags
Cell Ranger
STAR Solo
Median
WGS
Exome

Figure S7

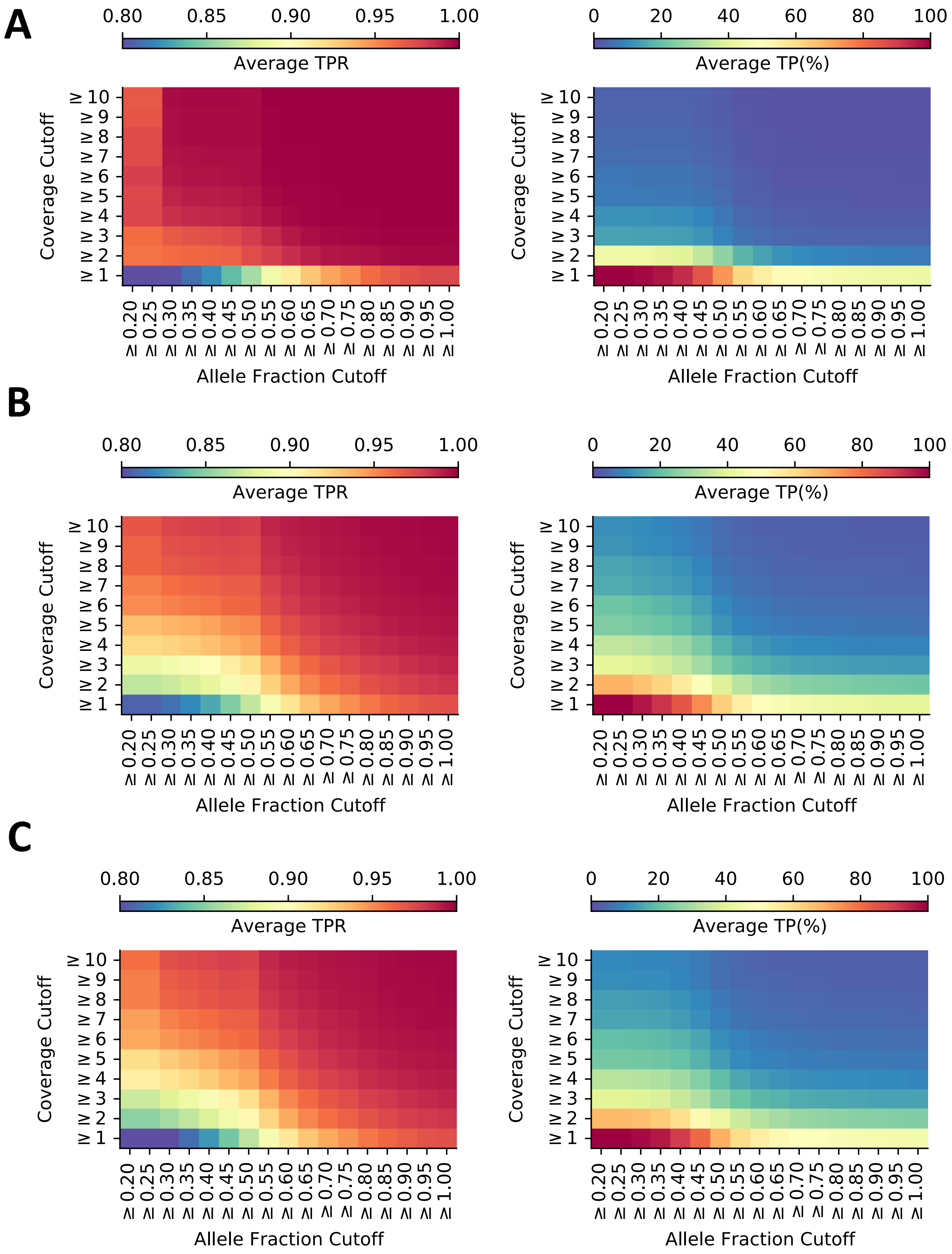
A
B
C
0.80
0.85
0.90
0.95
1.00
Average TPR
0
20
40
60
80
100
Average TP(%)
Coverage Cutoff
≥ 10
≥ 9
≥ 8
≥ 7
≥ 6
≥ 5
≥ 4
≥ 3
≥ 2
≥ 1
≥ 0.20
≥ 0.25
≥ 0.30
≥ 0.35
≥ 0.40
≥ 0.45
≥ 0.50
≥ 0.55
≥ 0.60
≥ 0.65
≥ 0.70
≥ 0.75
≥ 0.80
≥ 0.85
≥ 0.90
≥ 0.95
≥ 1.00
Allele Fraction Cutoff

Figure S8

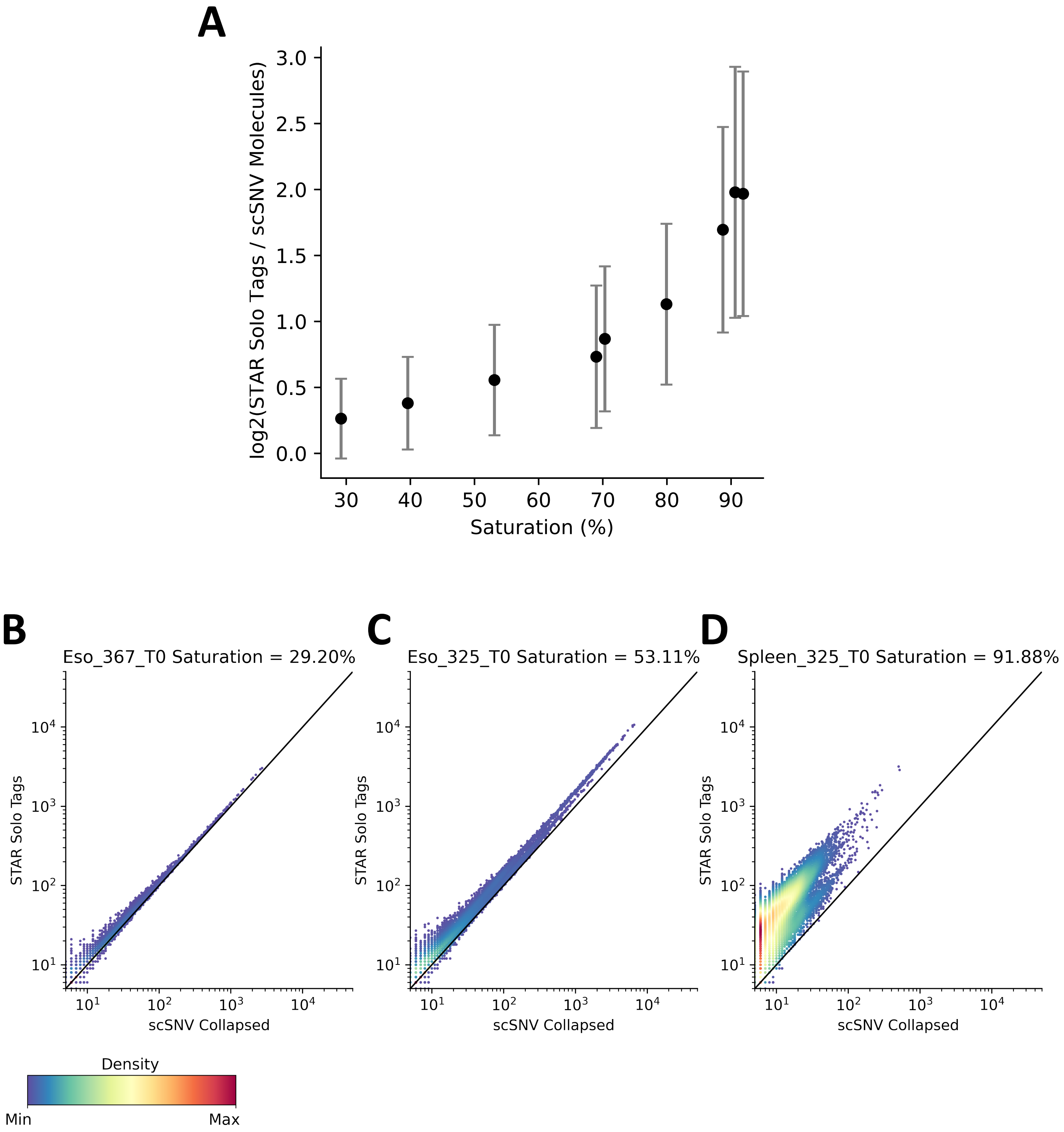
A
log2(STAR Solo Tags / scSNV Molecules)
3.0
2.5
2.0
1.5
1.0
0.5
0.0
30
40
50
60
70
80
90
Saturation (%)
B
Eso_367_T0 Saturation = 29.20%
C
Eso_325_T0 Saturation = 53.11%
D
Spleen_325_T0 Saturation = 91.88%
STAR Solo Tags
scSNV Collapsed
Density
Min
Max
