# Supplementary material for: scSNV: accurate dscRNA-seq SNV co-expression analysis using duplicate tag collapsing
Source: Genome Biol. 2021 May 7;22:144. doi: 10.1186/s13059-021-02364-5 (PMC8103760; doi:10.1186/s13059-021-02364-5)
Supplement: Supplementary file 3 — Additional file 3. Review history. [file 13059_2021_2364_MOESM3_ESM.docx]

Review History

**First round of review**

**Reviewer 1**

**Were you able to assess all statistics in the manuscript, including the appropriateness of statistical tests used?**

No

**Were you able to directly test the methods?**

No

**Comments to author:**

The manuscript entitled "scSNV: Accurate dscRNA-seq SNV co-expression analysis using duplicate tag collapsing" proposes a new SNV caller named scSNV for droplet-based single-cell RNA-sequencing. The authors are trying to reduce the false-positive discovery rate of SNV finding using collapsing Unique molecular identifiers (UMI) of duplicated reads and co-expression of SNV. For the evaluation of scSNV, they compared the tool and Cell Ranger (CR), which is most commonly used to analyze dscRNA-seq data with public data sets.

Major comments:

While the idea proposed in this paper using UMI collapse is interesting, it has an inherent shortcoming in performance evaluation. When benchmarking SNP callers, it is unclear whether they really improve performance, as several key metrics (F-score, specificity, sensitivity, etc.) that are usually required are not presented. It is also essential to generate simulation data and evaluate the performance of the proposed tool. Therefore, there is a lack of evidence that scSNV has reduced false positives compared to CR. Although CR has been able to find more SNVs than scSNVs, there should be more quantitative evidence that those SNVs are false positives.

The following paper may help conduct a performance evaluation of SNP caller:

https://genomebiology.biomedcentral.com/articles/10.1186/s13059-019-1863-4

Miner comments:

1. Please show why scSNV is faster than CR.

2. A number of SNP callers have already been published and applied to scRNA-seq. Can you run them after UMI collapse to compare them?

**Reviewer 2**

**Were you able to assess all statistics in the manuscript, including the appropriateness of statistical tests used?**

Yes: Several figures presented shows the percentages of different categories (eg Fig 2), the samples are not sorted by studies, making it difficult to assess inter-study differences; neither there is statistical test to show the difference among contigs of different length (Fig 2B); For another example, for binned sample comparison among SC vs CR methods, no statistical tests are done either.

**Were you able to directly test the methods?**

Yes

**Comments to author:**

This manuscript is about single nucleotide variant (SNV) pipeline called scSNV, optimized for droplet-based single-cell RNA-Seq data. The authors go into depth to handle the PCR duplication issues in the scRNA-Seq amplification, and design a pipeline that collapses the reads into longer units, upon which to be used for SNV detection. A tool to detect SNVs in scRNA-Seq is of general interest to the single cell genomics community. Below are my major and minor concerns of the work:

1. The claim of reduced false positive variant call rate by scSNV is not supported by the data/results. In Figure 3, Cell Ranger has a lot more SNVs of transition and transversion, compared to scSNV, so the authors claim that these are mostly false positive. This is speculation rather than fact, since there is no truth measure. Using simulation data will be a lot more convincing.

2. Cell Ranger is a commercial pipeline by 10x genomics, it has not versioning. This reviewer thinks the authors should compare their pipeline with open source methods, such as STAR or STAR solo.

3. The authors claimed that " Furthermore, a systematic evaluation of SNV calling accuracy from dscRNA-seq data has not been carried out. " (Page 9, lines 56-58) This is incorrect. Several comparison papers below have been published to address this issue (they should be cited). The methods mentioned in the paper should be compared (monoVar, varScan, samtools etc).

Systematic comparative analysis of single-nucleotide variant detection methods from single-cell RNA sequencing data, Genome Biol. 2019 Nov 19;20(1):242. doi: 10.1186/s13059-019-1863-4.

SNV identification from single-cell RNA sequencing data, Hum Mol Genet. 2019 Nov 1;28(21):3569-3583.

4. Intuitively, collapsing the reads to make longer "contigs" will be very beneficial for larger variations (eg. structural variations), rather than small variations (SNVs). Suggest the authors to investigate effect of their pipeline towards this end.

5. It will be interesting to specifically show the effect of removing 2nd PCR duplication. Does this increase or decrease the SNV accuracy?

6. Fig 6 does not appear to be fair comparison though, between scSNV vs cell ranger, since scSNV has longer collapsed reads, whereas cell ranger does not. It should be normalized by the same unit, saying, 100bp?

Minor comments:

1. Figure 5, p-values are missing in each pair of boxplots.

2. 5 esophageal adenocarcinoma organoid (EAC) samples and paired exome-seq data are not available. This does not help with reproducibility. Suggest to replace with public data (such as those in the reference of Hum Mol Genet. 2019 above), or make the data accessible by public.

**Authors Response**

**Point-by-point responses to the reviewers’ comments:**

*Detailed Rebuttal:*

*We first wish to thank both reviewers for their helpful feedback. Implementing their suggestions has greatly improved our manuscript. Our response to each individual comment is below:*

Review #1 Major Comment #1:

While the idea proposed in this paper using UMI collapse is interesting, it has an inherent shortcoming in performance evaluation. When benchmarking SNP callers, it is unclear whether they really improve performance, as several key metrics (F-score, specificity, sensitivity, etc.) that are usually required are not presented. It is also essential to generate simulation data and evaluate the performance of the proposed tool. Therefore, there is a lack of evidence that scSNV has reduced false positives compared to CR. Although CR has been able to find more SNVs than scSNVs, there should be more quantitative evidence that those SNVs are false positives.

*Response:*

*We agree with the reviewer that this was a deficiency in our analyses. However, given that a protocol does not presently exist for simulating dscRNA-seq data for SNV calling, we have developed an alternative strategy by using biological samples with matched DNA sequencing data as a gold standard to estimate the true positive rate, false discovery rate and F1-score. In our study, we had nine samples with matched WGS sequencing data and two with matched exome data. Our accuracy estimates were consistent with our previous finding. For example, our method had a similar true positive rate as Cell Ranger and STAR Solo (added to revisions) but a drastically reduced false discovery rate. We verified these results using two additional SNV calling methods, Strelka 2 and samtools/bcftools. Furthermore, we have rewritten the SNV calling sections to reflect this and have modified the introduction and discussion to reflect the same. For reproducibility, we have also included additional code in scSNV to extract similar accuracy data from samples with matched DNA sequencing data. Please see following revised results sections:*

*1. scSNV has fewer false positive SNV calls than Cell Ranger and STAR Solo*

*2. The majority of false positives detected by scSNV Pileup have low minor allele fractions*

*3. Exploring the effect of dscRNA-seq sparsity on the TPR, FDR and A>G edit detection and our revised conclusions.*

Review #1 Minor Comment #1:

Please show why scSNV is faster than CR.

*Response:*

*We have modified the text of our alignment performance section to describe the differences in run-time speed for scSNV, Cell Ranger and STAR Solo (Please see also Results section entitled: “Benchmarking the run time of scSNV and quantification accuracy versus Cell Ranger and STAR solo”.*

Review #1 Minor Comment #2:

A number of SNP callers have already been published and applied to scRNA-seq. Can you run them after UMI collapse to compare them?

*Response:*

*To compare additional SNV callers we used the two recommended methods from Liu et al. (doi.org/10.1186/s13059-019-1863-4), namely samtools/bcftools and Strelka2. We observed that Strelka2 had an overall poor performance on dscRNA-seq data and samtools/bcftools to exhibit a trade-off with our scSNV Pileup method.*

Review #2 Major Comment #1:

The claim of reduced false positive variant call rate by scSNV is not supported by the data/results. In Figure 3, Cell Ranger has a lot more SNVs of transition and transversion, compared to scSNV, so the authors claim that these are mostly false positive. This is speculation rather than fact, since there is no truth measure. Using simulation data will be a lot more convincing.?

*Response:*

*We agree with the Reviewer #2 and this was an issue brought up by Reviewer #1 as well, which we resolved using matched DNA sequencing (See our response above in Reviewer #1 Major Comment #1).*

Review #2 Major Comment #2:

Cell Ranger is a commercial pipeline by 10x genomics, it has not versioning. This reviewer thinks the authors should compare their pipeline with open source methods, such as STAR or STAR solo.

*Response:*

*The reviewer makes a valid point about Cell Ranger; therefore, to facilitate reproducibly, we have mirrored the source code for the exact version of Cell Ranger we used in our manuscript on our Github. Furthermore, we have also included STAR Solo in our analyses as a comparison.*

Review #2 Major Comment #3:

The authors claimed that " Furthermore, a systematic evaluation of SNV calling accuracy from dscRNA-seq data has not been carried out. " (Page 9, lines 56-58) This is incorrect. Several comparison papers below have been published to address this issue (they should be cited). The methods mentioned in the paper should be compared (monoVar, varScan, samtools etc).

Systematic comparative analysis of single-nucleotide variant detection methods from single-cell RNA sequencing data, Genome Biol. 2019 Nov 19;20(1):242. doi: 10.1186/s13059-019-1863-4.

SNV identification from single-cell RNA sequencing data, Hum Mol Genet. 2019 Nov 1;28(21):3569-3583.

*Response:*

*We did not include the aforementioned manuscripts originally as they focused on the Fluidigm C1 and SMART-seq based protocols; however, we have modified the section to include a reference to these methods. We have also expanded our analyses to include SNVs called using our scSNV Pileup method, Strelka2 and samtools/bcftools, (the latter two methods recommended by Genome Biol. 2019 Nov 19;20(1):242). Furthermore, we have modified our pileup method to work with a user provided list of SNV sites that can be utilised to extract strand- and cell- specific allele counts to facilitate the use of any available SNV caller on dscRNA-seq data.*

Review #2 Major Comment #4:

Intuitively, collapsing the reads to make longer "contigs" would be very beneficial for larger variations (eg. structural variations), rather than small variations (SNVs). Suggest the authors to investigate effect of their pipeline towards this end.

*Response:*

*We agree that UMI duplicate collapsing could be a useful method to identify larger structural variations and gene fusions; however, our alignment-based approach is less suitable to identify these. Therefore, we have modified our discussion mentioning that it may be possible to “assemble” the mRNA tags from reads derived from the same Cell Barcode and UMI and then mapping them to genome/transcriptome to identify potential SVs and fusions.*

Review #2 Major Comment #5:

It will be interesting to specifically show the effect of removing 2nd PCR duplication. Does this increase or decrease the SNV accuracy?

*Response:*

*To test this, we have included SNVs called using scSNV without collapsing but the PCR duplicates were discarded in our comparisons. We found the results were similar but slightly lower than scSNV with collapsing.*

Review #2 Major Comment #6:

Fig 6 does not appear to be fair comparison though, between scSNV vs cell ranger, since scSNV has longer collapsed reads, whereas cell ranger does not. It should be normalized by the same unit, saying, 100bp?

*Response:*

*The purpose of Figure 6 was to demonstrate that the collapsed reads can be used to identify additional co-expressed SNVs that would not be detected using mRNA tags alone. However, we have modified this figure to compare scSNV with and without read collapsing rather than Cell Ranger and STAR Solo as both latter methods are limited to 10 mismatches in 98 bp reads. BWA-mem, which we have used for our underlying alignment has a higher mismatch tolerance as reflected in the figure.*

Review #2 Minor Comment #1:

Figure 5, p-values are missing in each pair of boxplots

*Response:*

*We have replaced this figure with one comparing the TPR and FDR at the single cell level and have included p-values and R-squared values.*

Review #2 Minor Comment #2:

5 esophageal adenocarcinoma organoid (EAC) samples and paired exome-seq data are not available. This does not help with reproducibility. Suggest to replace with public data (such as those in the reference of Hum Mol Genet. 2019 above), or make the data accessible by public.

*Response:*

*We are happy to provide the raw data from the EAC samples upon request and the exome data is now published at (https://doi.org/10.1038/s41598-020-71589-4). We feel that the two samples with paired-exome data were helpful to our analyses as they were used to demonstrate TPR and FDR in regions constrained to those covered by exome. The datasets from the linked paper were generated with the Fluidigm C1, which does not have the two step-PCR necessary to generate collapsed reads.*

**Second round of review**

**Reviewer 1**

The authors have improved all the concerns pointed out by the reviewers. The manuscript would be suitable for publication in Genome Biology.

**Reviewer 2**

The authors have been largely responsive to the comments of the reviewer, such as adding benchmarking with STAR and STAR solo, as well as compare with bamtools and strelka2 SNP callers. The revision, however, still has some major concerns. Specifically:

1. The authors stated that there is not approved protocol for SNP based simulations in single cells, instead they opt to use the bulk exome-seq or WGS as the reference. As the result, the true positive rate is substantially low. Such a low value is a clear indication that their choice of “truth data” is not appropriate: in bulk samples, you do not know which single cells give rise to the variants. This has caused a lot of problems in the downstream analysis, reflected in Figure 3. One could alternatively use the bam files that they have in their current scRNA-Seq data, randomly introduce SNPs, and then run the “modified” reads through the pipelines in benchmarking.

2. I am very surprised that strelka2, a top method recommended by Liu etc. (Genome Biology 2019) performs so poorly (also no use) in Table 1. Did the authors contact Liu etc to discuss why there is such discrepancy in their results?

Reference: Liu F, Zhang Y, Zhang L, Li Z, Fang Q, Gao R, et al. Systematic comparative analysis of single-nucleotide variant detection methods from single-cell RNA sequencing data.

Genome Biology. 2019 Nov 19;20(1):242.

3. It sounds to me that the benefit of their pipeline lies in collapsing the reads as shown in Fig 4, however, the pileup strategy in their method is hurting rather than helping the SNPs calls, since pileup yields higher FDR compared to bamtools/vcftools.

4. The statement that "Data available upon request" can be read as possibly not providing data and this does not help with reproducibility of the results in the manuscript.

Minor point: Fig 6 needs statistical tests to state that the two approaches yields significant different outcome (rather than just numbers).

**Authors Response**

**Point-by-point responses to the reviewers’ comments:**

Detailed Rebuttal:

1. The authors stated that there is not approved protocol for SNP based simulations in single cells, instead they opt to use the bulk exome-seq or WGS as the reference. As the result, the true positive rate is substantially low. Such a low value is a clear indication that their choice of “truth data” is not appropriate: in bulk samples, you do not know which single cells give rise to the variants. This has caused a lot of problems in the downstream analysis, reflected in Figure 3. One could alternatively use the bam files that they have in their current scRNA-Seq data, randomly introduce SNPs, and then run the “modified” reads through the pipelines in benchmarking.

*Author Response: Thank you for your comment. We now see how the data as presented could be misinterpreted. The true positive rate we presented in Figure 4 was based on the full set of SNVs identified in the bulk sample rather than the SNVs positions that are expressed in a single-cell. Since 10X scRNA-seq data has thousands to tens-of-thousands of molecules per cell, we expect only a small subset of the true SNVs to be expressed in each cell, which substantially lowers the TPR.*

*Seeing as how the results we presented would be confusing, we have revised the section and created a new series of plots where for each cell we constrained the false negatives to only true positive SNVs from the bulk data that have at least 1X coverage in the given cell. This results in a much higher TPR value for each cell (median ~0.8). We have revised the results section and replaced figure 4 with this analysis and moved the original figure 4 to the supplement. Moreover, we created an additional panel for figure 4 (B and C) that demonstrates the sparsity of 10X scRNA-seq data where the majority of SNVs in a given cell are supported by a single read and this contributes to the reduction in the true positive rate when examining single cells.*

2. I am very surprised that strelka2, a top method recommended by Liu etc. (Genome Biology 2019) performs so poorly (also no use) in Table 1. Did the authors contact Liu etc to discuss why there is such discrepancy in their results?

*Author Response: The Liu paper almost exclusively studied SMART-seq2 scRNAseq technology and, as you know, these two technologies are quite different, and analysis of the resulting data is not interchangeable. SMART-seq2 generates paired-end reads of much higher coverage per cell, but of lower cellular throughput compared to 10X. 10X uses 3’ tag single-end sequencing to increase throughput but with much fewer reads per per cell (higher sparsity). We are therefore not particularly surprised that Strelka2, a method that was developed for paired-end RNAseq, also works well with SMART-seq2 but not with the single-end 3’-tag droplet scRNA-seq 10X data.*

*We further reviewed the Strelka2 documentation and found that it is not recommended on samples that do not have paired-end RNA-seq. Indeed, we checked the raw unfiltered vcf files generated from Strelka2 and verified that the majority of SNV calls were filtered due to their genotype quality filter. We feel this is important as it demonstrates that it is not an appropriate tool for 10X scRNA-seq data.*

*We further examined the Liu paper and their section that explored 10X dscRNA-seq data was brief. Furthermore, their methods and results did not explain why they had so few cells, 78 from their main text when the Wang et al., preprint from which they took their cells indicates that they had 1,338 cells in their hepatocellular carcinoma dataset. The authors also do not indicate how they called SNVs on the 10X dataset as Cell Ranger outputs a BAM file with the alignments from all the cells pooled together. If the authors extracted 78 random barcodes from the bam file and processed each of those individually as they did for their SMART-seq2 cells, the library size would be 10,000 to 100,000 reads prior to mapping and duplicate removal as indicated in Wang et. We doubt that this SNV callers would function correctly on a single cell with such low read depth, as we have shown in Figure 4C that the many of SNVs in a given 10X have supporting 1-2 tag alignments /molecules.*

*Nonetheless, we attempted to re-create Liu et al.’s Figure 4D using the same high confidence protein coding regions as their paper and our 9 normal and 2 esophageal organoid samples. We used Cell Ranger for Alignment, which is what Liu et al. used for their comparisons. We did not apply the 0.25 allele fraction cut-off we chose in our paper. The maximum number of SNVs in high confidence regions for each alignment method were as follows: scSNV pileup, 848, samtools/bcftools, 352, and strelka2, 96. The median number of SNVs ranged from scSNV pileup, 33-92, samtools/bcftools, 17-74, and Strelka2, 8-33 (see the top row of the figure below). While the Liu et al. paper identified 100-10,000 SNVs per cell depending on the tool, with a median of approximately 700-1,000.*

*For comparison, we used all of the SNVs without the high confidence region constraint. We found the maximum number of SNVs in a cell to be scSNV pileup, 8,828, samtools/bcftools, 5,320, and Strelka2, 518. The median number of SNVs ranged from scSNV pileup, 373-1163, samtools/bcftools, 200-741, and strelka2, 41.5-120 (see the bottom row of the figure below). These numbers are more consistent with what Liu et al. have shown in Figure 4D.*

*We do not doubt that Liu et al. are correct that Strelka2 performs well with full-length scRNA-seq data, however, we caution the interpretation of their 10X results given the discrepancy between the number of cells they used in their manuscript and the number of SNVs they detected being much higher than what we observed with Cell Ranger and samtools/bcftools which was also used by the authors in their manuscript. Nonetheless, we have revised our results for Strelka2 to indicate that the majority of true positive SNV calls were marked as low quality by their variant filtration algorithm. We do not feel that adding the analysis above adds to our manuscript but would be happy to do so at the discretion of the editor.*

3. It sounds to me that the benefit of their pipeline lies in collapsing the reads as shown in Fig 4, however, the pileup strategy in their method is hurting rather than helping the SNPs calls, since pileup yields higher FDR compared to bamtools/vcftools.

*Author Response: We discussed this extensively in our discussion. Our scSNV Pileup does indeed have a higher false positive rate than samtools/bcftools, however, scSNV Pileup has a slightly higher true positive rate, detects thousands of additional A-to-G edits and would be useful if the SNVs a user was interested in had an allele fraction lower than ~20%, which appears to be threshold of detect for samtools/bcftools with the default parameters. Moreover, we developed scSNV pileup to facilitate the extraction of single cell SNV count data and other metrics from a pooled BAM file using SNVs called using other methods as tools such as samtools/bcftools do not currently provide this functionality.*

4. The statement that "Data available upon request" can be read as possibly not providing data and this does not help with reproducibility of the results in the manuscript.

*Author Response: Our analysis of the EAC patient samples focus on germline SNV calling and can theoretically be used to identify a specific patient. Our REB therefore does not allow us to publicly release the data, but we will consider data sharing if an institutional MTA is signed (which is common for many datasets). We believe it is our obligation as authors on the manuscript to share the data if appropriately requested. Most of the data is already in the public domain so this only affects the few esophageal cancer samples derived from our patients.*

Minor point: Fig 6 needs statistical tests to state that the two approaches yields significant different outcome (rather than just numbers).

*Author Response: We thank the reviewer for pointing this out and have added a statistical test to the text of the pertinent results section.*

**Third round of review**

**Authors Response**

**Point-by-point responses to the reviewers’ comments:**

*Rebuttal:*

*1) The necessity to have simulated data in our benchmarking studies*

*We agree with both reviewers that a simulation experiment was necessary to demonstrate that our lower-than-expected true positive rates (TPR) using matched genotyping data at the single cell level were due to the sparsity of dscRNA-seq data rather than an issue with the alignment and SNV calling algorithms used in this manuscript. To this end, we have included three simulation analyses, where we used a previously described simulation method (https://doi.org/10.1186/s13059-019-1863-4) to introduce 25,000 random homozygous SNVs in the reference genome at positions constrained to having at least 5X coverage by scSNV with collapsing and STAR Solo. The limitation of this method is that only clonal homozygous variants are generated from pre-existing reads from a biological sample, as a protocol to simulate dscRNA-seq reads currently does not exist. Nonetheless, this limitation resolves the issues brought up by Reviewer #2 in that we cannot be sure if a heterozygous SNV is not present in a cell due to the sparsity of dscRNA-seq or because it is a subclonal mutation. For our simulated data, if a simulated loci is expressed in a given cell, the SNV should be detected, with the exception of alignment of SNV calling issues.*

*We generated an independent reference genome for all three different droplet-based single cell RNA-seq experiments (dscRNA-seq) and detected more than 90-96% of the 25,000 simulated SNVs with scSNV or STAR Solo using scSNV Pileup or samtools/bcftools for SNV calling. At the single-cell level we constrained our analyses to only those regions with expression in a given cell and observed a median TPR greater than 0.94. This demonstrates that if a SNV is expected to be detected and there is expression at its loci, in a given cell, it will generally be detected by any of the methods used in our manuscript. The former point is critical to the analyses of dscRNA-seq data and we have substantially revised our manuscript to describe the typical SNV calling process for a dscRNA-seq sample.*

*Finally, we have revised our manuscript to better describe that SNVs are called on dscRNA-seq samples using a pseudo-bulk approach where all of the cells are aggregated together as the sparsity of the data and the computational complexity of extracting the reads for thousands or tens of thousands of single-cells and processing them individually is unnecessary.*
